# Silencing efficiency of dsRNA fragments targeting *Fusarium graminearum TRI6* and patterns of small interfering RNA associated with reduced virulence and mycotoxin production

**DOI:** 10.1371/journal.pone.0202798

**Published:** 2018-08-30

**Authors:** Thomas Baldwin, Emir Islamovic, Kathy Klos, Paul Schwartz, James Gillespie, Samuel Hunter, Phil Bregitzer

**Affiliations:** 1 National Small Grains Germplasm Research Facility, USDA-ARS, Aberdeen, Idaho, United States of America; 2 BASF, Research Triangle Park, NC, United States of America; 3 Department of Plant Sciences, North Dakota State University, Fargo, ND, United States of America; 4 iBEST, University of Idaho, Moscow, ID, United States of America; University of Nebraska-Lincoln, UNITED STATES

## Abstract

Deoxynivalenol (DON) contamination of cereal grains caused by Fusarium head blight may be addressed by future RNA interference (RNAi)-based gene silencing approaches. However, utilizing these approaches will require a greater understanding of the principles that govern RNAi effectiveness in the pathogen *Fusarium graminearum*. RNAi in higher eukaryotes, including fungi, involves processing double stranded RNA (dsRNA) into small interfering RNA (siRNA) that silence gene expression based on base pair complementarity. This study examined virulence, DON production, and the small RNA (sRNA) populations in response to RNAi-based silencing of *TRI6*, a transcription factor that positively regulates DON synthesis via control of *TRI5* expression. Silencing was accomplished via the expression of transgenes encoding inverted repeats targeting various regions of *TRI6* (RNAi vectors). Transgene expression was associated with novel, *TRI6-*specific siRNAs. For RNAi vectors targeting the majority of *TRI6* sequence (~600 bp), a discontinuous, repeatable pattern was observed in which most siRNAs mapped to specific regions of *TRI6*. Targeting shorter regions (250–350 bp) did not alter the siRNA populations corresponding to that region of *TRI6*. No phased processing was observed. The 5' base of ~83% of siRNAs was uracil, consistent with DICER processing and ARGONAUTE binding preferences for siRNA. Mutant lines showed *TRI6* siRNA-associated reductions of *TRI5* expression on toxin inducing media and DON in infected wheat and barley spikes. Shorter RNAi vectors resulted in variable levels of silencing that were less than for the ~600 bp RNAi vector, with a 343 bp RNAi vector targeting the 5’ end of *TRI6* having the best silencing efficiency. This work identifies efficient shorter region for silencing of *TRI6* and describes the patterns of siRNA corresponding to those regions.

## Introduction

Fusarium head blight (FHB) of wheat and barley and kernel rot on corn, caused by *Fusarium graminearum*, is of economic and health importance globally [1, 2, 3, 4, 5]. Heavily blighted crops exhibit loss of grain yield, and quality is reduced by contamination with mycotoxins that affect human and animal health [1, 6, 7]. Multiple mycotoxins are produced by *F*. *graminearum*, the most prevalent being deoxynivalenol (DON) and other trichothecenes, including nivalenol and DON derivatives 3- and 15-acetyldeoxynivalenol [8, 9, 10]. Traditional approaches to breeding for FHB resistance and current management strategies have been only partially effective in limiting the impact of FHB. Recently, reduced pathogenicity was demonstrated via RNA interference (RNAi)-based host-induced gene silencing (HIGS) in which host plants expressed RNAi vectors encoding double stranded (ds) RNA that targeted key fungal genes [11, 12, 13, 14].

RNAi is an ancient cellular process where non-coding small RNA (sRNA) molecules, including small interfering RNA (siRNA) and microRNA (miRNA), guide the RNA-induced silencing complex (RISC) to complementary mRNA, resulting in mRNA cleavage or translational repression [15, 16]. siRNA and miRNA are typically 21–24 nt long and double-stranded, with 2–3 nucleotide 3’ overhangs due to their processing from longer double-stranded RNA (dsRNA) by the RNase III-like enzyme called DICER [17, 18, 19]. Incorporation of siRNA and miRNA into the RISC, which includes the endonucleolytic protein, ARGONAUTE, results in sequence-based targeting of specific mRNA species [15]. Recently, the discovery of two genes encoding the *F*. *graminearum* DICER and ARGONAUTE homologs, *FgDICER2 and FgAgo1*, led Chen et al. (2015) to propose that they were critical for processing transgene-derived dsRNA in this species.

Researchers have utilized RNAi silencing to study the function of fungal genes by introducing constructs that encode inverted repeats of a target gene and this was found to be more effective than introducing a single reverse strand of the target gene [20, 21]. Transcripts containing inverted repeats form dsRNA as the single stranded RNA folds back onto itself, creating a stem-loop structure. The dsRNA portion becomes a target for DICER processing (reviewed in [22]). McDonald et al. (2005) transformed *F*. *graminearum* to express an inverted repeat of a slightly truncated *TRI6*. TRI6 is a transcription factor that positively regulates production of trichothecene mycotoxins (including DON) by regulating expression of the TRI genes in the biosynthetic pathway [23, 24]. This includes *TRI5*, which encodes the enzyme involved in the first step in the synthesis of trichothecene mycotoxins [10]. *TRI6* RNAi-expressing strains were less able to spread within the wheat head, a process that is facilitated by DON, and grain from infected spikes did not contain detectable levels of DON. Presumably, this was mediated by processing of the ds*TRI6* mRNA, leading to reduced expression of *TRI6* and *TRI5*, and consequently a reduction in the innate ability of the RNAi mutant strains to produce DON. Later, transformation with pTRM-TRI6 was shown to reduce *TRI5* expression and DON production *in vitro* in *F*. *culmorum* [25]. However, neither study showed evidence that siRNA production from ds*TRI6* RNA had occurred.

The gene expression control by RNAi in fungi, which are reviewed above, involve the expression of dsRNAs that are processed by DICER into populations of siRNAs. Not all sRNAs (including siRNAs and miRNAs) are equivalent with respect to their silencing potential [16, 26, 27]. Furthermore, the specific gene sequence targeted by sRNAs can influence the degree of silencing [28, 29]. However, prior reports of successful RNAi vectors present only the results of vectors that successfully induced silencing of targeted genes, without systematically investigating how targeting specific sequences influence silencing, although the work on grape root inhibition of root-knot nematode suggested that this might be important [30]. Furthermore, detailed data on the siRNA populations resulting from the expression of RNAi vectors is lacking.

This study 1) investigated whether the phenotypes that resulted from transformation with pTRM-TRI6 were a result of RNAi-mediated downregulation of DON synthesis; 2) determined the silencing efficacies of RNAi vectors targeting various regions of *TRI6;* and 3) characterized the siRNA populations derived from expression of these vectors in *F*. *graminearum*.

## Materials and methods

### Strains and growth conditions

The wild type *F*. *graminearum* strain PH1 (NRRL 31084, FGSC 9075) was used for experiments as a control and background strain for transformation. All strains were grown and maintained on V8 media (see [31]) in 100 x 15-mm Petri plates. Mycelial growth assays and observations of mutants were performed on potato dextrose agar (Sigma-Aldrich, St. Louis, MO). For the production of conidia, agar plugs of 3-d-old cultures on V8 media were taken from actively growing mycelia along the periphery of the cultures and used to inoculate 100 mL of carboxymethyl cellulose (CMC) media [32] in 250 mL-Erlenmeyer flasks, and incubated for 4–5 d at 25°C on a rotary shaker at 150 rpm. Conidia were isolated by centrifugation at 2500 x *g*, followed by resuspension in 1 mL of sterile deionized H_2_O. Spore concentrations were determined using a hemocytometer. To obtain RNA under toxin inducing conditions, strains were grown on putrescine toxin induction media [32].

### RNAi vectors and protoplast transformation

The plasmids pTRM-TRI6 [33] and pUCH2-8 [34] encoding an inverted repeat with homology to ~610 bp of *TRI6* (total length of the *TRI6* coding region is 657 bp), and hygromycin resistance, respectively, were provided by S. McCormick (USDA-ARS, Peoria, IL) (Fig A in S1 File). PEG-mediated transformation of protoplasts was performed as described in [32] with 7 μg of pTRM-TRI6 and 4 μg of pUCH2-8 that had been linearized with EcoRI or SmaI, respectively. Transformants were selected on V8 medium amended with 150 μg g^-1^ hygromycin. See Table A and Figs A-F in S1 File for a list of primers used and for details of analysis of pTRM-TRI6 mutants.

For initial experiments, the vectors used for co-transformation with pTRM-TRI6 [33] and pUCH2-8 resulted in insertions into random locations. To avoid potential confounding effects of position effects on transgene insertion site and/or the effects of unintended insertions into native genes (Table B in S1 File), two vectors were designed to enable site-directed insertion by homologous recombination. All plasmids containing these homologous recombination direct insertion-sites are denoted pHR1-IIP or pHR2-IIP. Two sites were chosen, one inside (HR1) and one directly outside (HR2) of the TRI gene cluster on chromosome 2. These vectors included a hygromycin resistance cassette (Fig G in S1 File). Vectors containing the near full length *TRI6* inverted repeat cassette (described above) were used to create three mutant strains at each HR insertion site. Details of vector construction are in Fig G, Fig H, and Procedure A in S1 File.

To investigate the effects of targeting shorter regions of *TRI6*, five vectors were created that encoded inverted repeats of 250–350 bp with homology to five different segments of *TRI6*. These vectors were all based on the pHR2-IIp backbones. Details of vector assembly are in Fig I in S1 File.

All plasmids with the pHR1-IIP and pHR2-IIP backbone were restricted with I-SceI for linearization before transformation [35]. Transformants were selected on V8 medium amended with 150 μg g^-1^ hygromycin. The primer pair gfp frag-qPCR fw/rv (Table A in S1 File) was used to select transformants containing the TRI6 RNAi vectors.

### Whole genome Illumina-sequencing and analysis for random transgenic site integration mutants

DNA was extracted from pTRM-TRI6 mutants 1 to 6, puch2-8 mutants 1 to 3, and PH1 that were grown on liquid YEPD medium for 3 d on a rotator-shaker at 125 rpm (see [31]) and the mycelia were harvested by filtration with Whatman No 4 filter paper (Sigma-Aldrich, St. Louis, MO) a Büchner funnel under vacuum, rinsing with sterile ddH_2_O, and scraping the mycelia from the filter paper. Mycelia were ground in liquid N_2_ with a mortar and pestle. 100 mg of frozen, powdered mycelia was used for DNA extraction using the Norgen Biotek ™ DNA extraction kit (Thorold, Ontario, Canada). The quality of DNA was assessed on a Fragment Analyzer™ instrument (Advanced Analytical, Ankeny, IA) before whole genome sequencing. Genomic DNA was fragmented on the Bioruptor Plus UCD-300 (Diagenode, Denville, NJ) sonication system with six cycles of 30 sec on (low power setting) and 90 sec off in an ice water bath. For each strain (mutant or wildtype), libraries were prepared with the NEBNext Ultra DNA library prep kit (New England Biolabs, Ipswich, MS). Next generation sequencing (NGS) was performed on an Illumina MiSeq with the Illumina V3 reagents (Illumina, San Diego, CA) following the manufacturer's protocol. Strains were sequenced on two flow cells (five strains per flow cell) to attain between 3,000,000 to 6,000,000 reads per strain. Reads were aligned to the *F*. *graminearum* TRI cluster (Chromosome 2:5364908–5405224 retrieved from fungi.ensembl.org, [36]) to check for potential gene disruption. Illumina reads were aligned to the pTRM-TRI6 plasmid to capture chimeric reads with both plasmid and fungal genomic DNA sequence (originating from pTRM-TRI6/fungal junctions) to determine the genomic location of TRM-TRI6 insertions in each mutant. Alignment was performed with Geneious software version 8.1.4 (Biomatters, Ltd., Auckland, New Zealand) see Procedure A in S1 File for more detail.

### qPCR and endpoint PCR to determine copy number and confirm integration

Estimation of copy number was determined by qPCR in a single experiment. There were three technical replicates of the target and reference genes for each RNAi mutant strain. These assays used the primer pair "gfp frag-qPCR fw/rv” (E = 103.5%, R^2^ = 0.997) (Table A in S1 File). Comparisons were made to the single-copy β -tubulin gene that was amplified with primer pair “TubQ F/R” (E = 106.3, R^2^ = 0.982) (Table A in S1 File) using the ^ΔΔ^CT method and the CFX Manager Software package (Bio-Rad Laboratories, Hercules, CA). SsoFast EvaGreen Supermix (Bio-Rad) with the Sybrgreen fluorophore was used in this analysis with 25 ng of template genomic DNA and 500 nM of primer. Samples were processed in a CFX Connect thermocyler (Bio-Rad). The thermal cycler program was the following: 95° C for 30 seconds (s), 40 cycles of a melting step at 95° C for 5 s and an annealing/extension step at 60° C for 5 s.

The NGS-predicted genomic sites of random pTRM-TRI6 insertions were checked by endpoint PCR (from genomic DNA for pTRM-TRI6 mutant strains and PH1). The thermal cycler program was 98°C for 1 minute followed by 30 cycles of 98° C for 15 s: 55°C for 30 s: 72°C for 30s using Phusion polymerase (New England Biolabs). PCR primers in the transgene insert and flanking genomic DNA were designed for each predicted insertion site (Table A in S1 File) and checked for disruption by the insertion.

### qRT-PCR to determine *TRI5* expression

RNA was extracted from mycelium grown on putrescine toxin-induction medium and harvested 3 days post-inoculation (dpi). Mycelia were harvested as described above, and ground to a fine powder using a SPEX freezer mill (Spex Industries Inc., Edison, NJ) cooled with liquid nitrogen. A 1 min grind at a 10 cycles per second rate was used to grind the harvested mycelium. RNA was extracted from 100 mg of frozen powder using the GeneJET Plant Purification Minikit (Thermo Fisher Scientific, Waltham, MA, USA). Samples were then treated with DNase I and DNase 5x buffer (New England Biolabs). The RNA was then isolated via LiCl precipitation, and the iScript cDNA synthesis kit (Bio-Rad) was used for cDNA synthesis.

Estimation of *TRI5* expression was determined for PH1 and mutant strains in three experiments, each with three technical replicates for the target gene and the reference gene. Comparisons were made using the ^ΔΔ^CT method using the CFX Manager Software package (Bio-Rad Laboratories). Log_2_ transformations of the data were used to visualize changes in gene expression. Expression was normalized to wild-type (PH1) and the overall standard error was calculated as √(SE^2^[mutant^ΔCT^] + SE^2^[wt^ΔCT^]). *TRI5* was amplified using primers Tri5QF and Tri5QR (E = 112.0, R^2^ = 1.000) and the reference gene *TUB2* was amplified using the primersr TubQF/R (E = 106.3, R^2^ = 0.982) (Table A in S1 File). The standard protocol for SsoFast EvaGreen Supermix (Bio-Rad, Hercules, CA, USA) with the Sybrgreen fluorophore was followed using 0.5 μM of primer, and 25 ng of template cDNA. The thermal cycler program was 95°C for 30 s followed by 40 cycles of 95°C for 5 s: 60°C for 5 s. A CFX machine (Bio-Rad, Hercules, CA, USA) was used to perform the real-time PCR cycles.

### Virulence assays

Virulence on wheat was determined in three experiments. Wheat cultivar Klasic was planted in “Cone-tainers” (4-cm diameter × 20.5-cm long; Stuewe & Sons, Tangent, OR, USA) filled with a vermiculite/peat moss/sand mixture in a 1:1:1 ratio by volume and Osmocote 15-9-12 slow release fertilizer (25g/L; Scotts, Marysville, OH, USA). Plants were grown in growth chambers under fluorescent and incandescent lights with a 16-h photoperiod at temperatures of 14°C (dark period) and 4 h at 16°C, 8 h at 20°C, and 4 h at 16°C (light period, 54–90 μmol m^-2^). Inoculation was performed as described in Hallen-Adams et al. (2011). In brief, for each mutant strain, 10 μL of a solution composed of 5 x 10^5^ conidia/ml was injected with a syringe and needle into a single floret in each of three spikes per cultivar just prior to anthesis. The florets that were inoculated were 4 to 5 florets from the base of each spike. The spike was covered with a plastic bag for 3 d which was subsequently replaced with a glassine bag (L404, Lawson Northfield, IL) Wheat spikes were harvested 14 dpi, dried in a paper bag for 3 d at 37°C, and pulverized to a powder for DON analysis using a SPEX freezer mill as described above. Spikes were ground and sampled individually by experiment.

Virulence on barley was determined in a single experiment. Seven heads per strain were dip-inoculated (described below). Barley (*Hordeum vulgare*), cultivar Golden Promise, was grown as described for wheat, except that for the first 7 weeks the plants were grown in a greenhouse with diurnal temperatures from 12°C to 25°C and a ~14 h photoperiod (artificial light added below 40 μmol m^-2^ s^-1^). Inoculation of barley heads was performed with significant modifications from [31]. Instead of spraying a spore suspension onto spikes, whole spikes were immersed in a spore suspension (10^5^ conidia/mL) containing 4% Tween 20. Inoculated spikes were covered with a plastic bag for 3 d which was subsequently replaced with a glycine bag. Barley heads were harvested 21 dpi. Harvested heads were dried in a paper bag for 3 d at 37°C. For determination of the weight of infected florets, infected florets were removed by hand from spikes and weighed. Subsequently, infected florets were pulverized to a powder using a SPEX freezer mill as described above, and used for DON analysis. Heads by treatment were combined for analysis.

### GC-MS DON analysis

DON analysis was performed at North Dakota State University, Institute Of Barley And Malt Sciences. Analyses were as described in [37] In brief, either 500 mg or 1 g of pulverized infected tissues was extracted with 10 mL of 84% acetonitrile 16% water mixture, filtered, and diluted for sample analysis on a GC-MS. Concentration was adjusted per 1 g of sample.

### Small RNA sequencing

RNA was extracted from mycelium grown on putrescine toxin-induction medium using the same protocol outlined above for qRT-PCR, but without DNase I treatment. RNA samples were analyzed for integrity using either an Advanced Analytical Fragment Analyzer or an Agilent Bioanalyzer. RNA samples with RQN (RIN) scores >9 were accepted for sequencing. The 15–30 nt sRNA fraction was gel extracted to exclude genomic DNA and ribosomal RNA from sequencing as part of the Illumina TruSeq protocol. cDNA libraries of the sRNA fraction were prepared using the Illumina TruSeq Small RNA Library Preparation Kit (Illumina, San Diego, CA) with strict adherence to the manual. Briefly, the 3′ and 5′ RNA adapters were ligated on to fragments before a reverse transcription reaction was performed to synthesize cDNA. The cDNA library was further amplified with a PCR reaction. Next generation sequencing was performed on a MiSeq (Illumina) across multiple (2 or more) flow cells so that conditions and concentrations could be adjusted, if necessary, to achieve adequate read-depth. This is necessary due to the difficulty of adequately estimating the concentrations of sRNA preparations. All data files were uploaded to NCBI Bioprojects Sequence Read Archive at https://www.ncbi.nlm.nih.gov/bioproject/PRJNA446058

### Small RNA analysis

Rarefaction analysis was performed with 11 million reads from a single flow cell from a preliminary RNA sample from pTRM-TRI6 #1 using Illumina BaseSpace (https://basespace.illumina.com/home/index) and plotting reads to randomly selected sRNAs in Microsoft Excel. Low-quality reads were removed by trimming the raw sRNA reads using the CLC Genomics Workbench 9.0.1 (CLC bio, Arhus, Denmark) software modified Mott trimming algorithm (comparable to Phred score processing; Ewing et al. 1998) which evaluates sequences based on Illumina quality scores. Calls that did not meet a cumulative error probability threshold of 0.001 were discarded. As a result, no individual base pair had a Phred score below 30. Sequences above 30 nt and below 18 nt were also discarded using the same software. Alignment of sRNA reads to *TRI6* was accomplished in CLC Genomics Workbench using the “map reads to reference” function under default conditions (match score = 1, mismatch cost = 2, cost of insertion and deletion = linear gap cost, insertion cost = 3, deletion cost = 3, length of fraction = 0.5, similarity fraction = 0.8, color error cost = 3). The mapped reads were exported as a SAM file, and the sense and antisense reads were separated using a python script written by S. Hunter (University of Idaho IBEST Genomic Research Core facility). Reads were re-aligned to *TRI6* in CLC Genomics Workbench and read numbers per nucleotide were exported into a comma separated values file and graphed in Microsoft Excel. This process was performed for individual technical reads, pooled technical reads, and all pooled reads from mutants.

## 2. Results

### 2.1 Mutant strain production and characterization

A total of 16 hygromycin-resistant transformants were recovered from co-transformation with pTRM-TRI6 and pUCH2-8 (see supplementary information for details). Ten were positive for pTRM-TRI6, and all were positive for pUCH2-8, as determined by PCR. Six pTRM-TRI6 mutant strains were selected at random from the pool of mutants and characterized for the number and chromosomal location of transgene insertions. For all strains, the native *TRI* genes were shown to be intact and uninterrupted by the transgene insertions. Three pTRM-TRI6 negative, pUCH2-8 positive mutant strains were included as controls to assess the effects of hygromycin expression. All six of the selected mutant strains were used in the analysis of virulence and DON production. pTRM-TRI6 mutant strains 1, 4, and 5 were used for the sRNA analysis.

Mutants containing *TRI6* RNAi vectors targeting region HR2, located just outside the TRI cluster, were selected for on hygromycin. Six to 10 unique transformants were obtained per vector. All hygromycin resistant transformants contained the expected inverted repeat sequence, as determined by PCR amplicon size (via amplification from the GFP fragment loop into the inverted repeat-specific sequence). All insertions were into the HR2 region as expected based on the homologous recombination method used for insertion. For each HR *TRI6* RNAi vector three mutants were assayed and represented in virulence and qRT-PCR experiments. Small RNA analysis was performed on four full-length HR RNAi mutants. Only one mutant for each of the five short RNAi vectors was assayed. Strains are described in Table 1 and can be requested through the database FgMutantDb (scabusa.org/fgmutantdb, [38]).

**Table 1:** RNAi mutant strains used in this study.

RNAi mutant strain	NCBI SRA Accession^a^	RNAi fragment length	Start^b^	End^b^	Type of Insertion
pTRM-TRI6-1	SRX3857032	607 bp	-33	-640	Random
pTRM-TRI6-4	SRX3857031	607 bp	-33	-640	Random
pTRM-TRI6-5	SRX3857030	607 bp	-33	-640	Random
HR1-IIP-TRI6IR-1	SRX3857029	607 bp	-33	-640	site-directed
HR1-IIP-TRI6IR-2	SRX3857036	607 bp	-33	-640	site-directed
HR2-IIP-TRI6IR-1	SRX3857035	607 bp	-33	-640	site-directed
HR2-IIP-TRI6IR-2	SRX3857034	607 bp	-33	-640	site-directed
HR2-IIP-ShortTRI6IR-1	SRX3857033	253 bp	0	-253	site-directed
HR2-IIP-ShortTRI6IR-2	SRX3857038	256 bp	-186	-443	site-directed
HR2-IIP-ShortTRI6IR-3	SRX3857037	266 bp	-406	-672	site-directed
HR2-IIP-ShortTRI6IR-4	SRX3857040	342 bp	0	-342	site-directed
HR2-IIP-ShortTRI6IR-5	SRX3857039	323 bp	-349	-672	site-directed

^a^ NCBI Bioprojects Sequence Read Archive project number PRJNA446058

^b^Start and end position based on 5’ end of the *TRI6* CDS.

### 2.2 *TRI5* and DON suppression in RNAi mutant strains and effects on virulence

**Fig 1:**
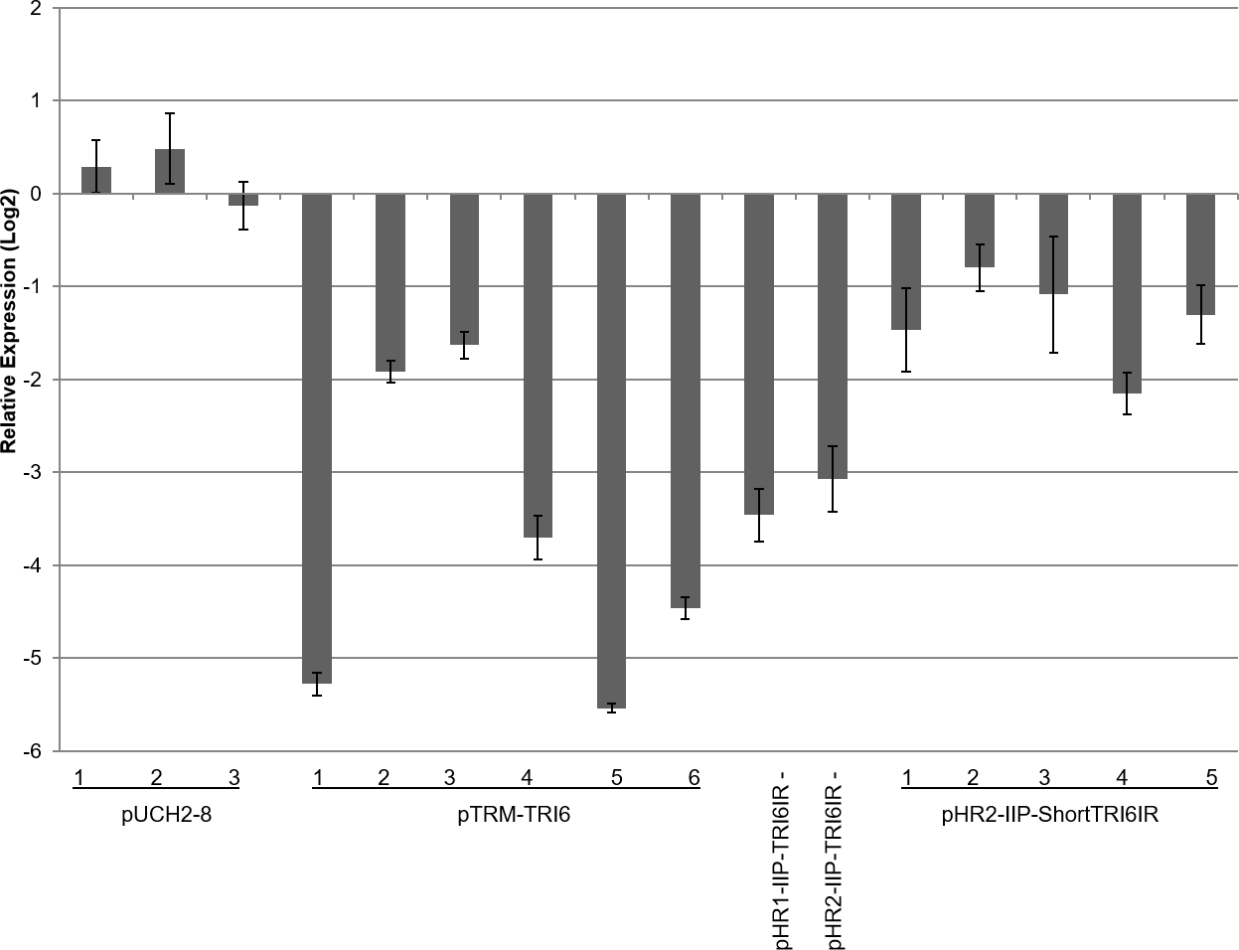
Expression of *TRI5* via qRT-PCR in PH1, and in pUCH2-8 and *TRI6*-RNAi mutant strains (full-length and short length) grown on toxin induction media. The Log_2_ expression is normalized to PH1. Line bars denote SE of three experiments.

Characterization of *TRI6* RNAi expression via qRT-PCR is hampered by the difficulty of amplifying inverted repeat sequences, and because its expression cannot be distinguished from expression of native *TRI6*. Therefore, the expression was inferred based on the expression of the *TRI6*-regulated *TRI5* in mycelium cultured on putrescine toxin-induction media. *TRI5* expression 3 dpi showed an average 2.65 log_2_ fold decrease (Fig 1) in pTRM-TRI6 RNAi mutant lines versus PH1 and the pUCH2-8 (hygromycin-resistance only) control mutant strains. Note that although each of these lines had identical IR transgenes, the degree of silencing varied (range = 1.6- to 5.5-fold decrease). The site-directed full-length *TRI6* mutants showed similar responses (a 3.4 and 3.0 log_2_ fold decrease in *TRI5* expression for HR1 and HR2, respectively). The short fragment RNAi mutant lines showed smaller decreases (0.79–2.15 log_2_ fold) than the full-length lines, with short *TRI6* fragment 2 silencing the least (0.79 log_2_ fold) and short *TRI6* fragment 4 silencing the most (2.15 log_2_ fold), testing 3 independent mutants for each line with 3 technical replications.

To investigate their innate DON-producing capacities in the absence of the host-pathogen interaction, the mutant strains and PH1 were used to inoculate rice cultures composed of autoclaved, commercially-available white rice (Fig J in S1 File). These assays showed that cultures inoculated with PH1 contained 52.5 μg g^-1^ DON, and that the DON concentrations of the pUCH2-8 cultures ranged from 54.3 to 97.3 μg g^-1^. In contrast, cultures inoculated with pTRM-TRI6 mutant strains showed marked reductions in DON concentrations (0.05–5.5 μg g^-1^).

**Fig 2:**
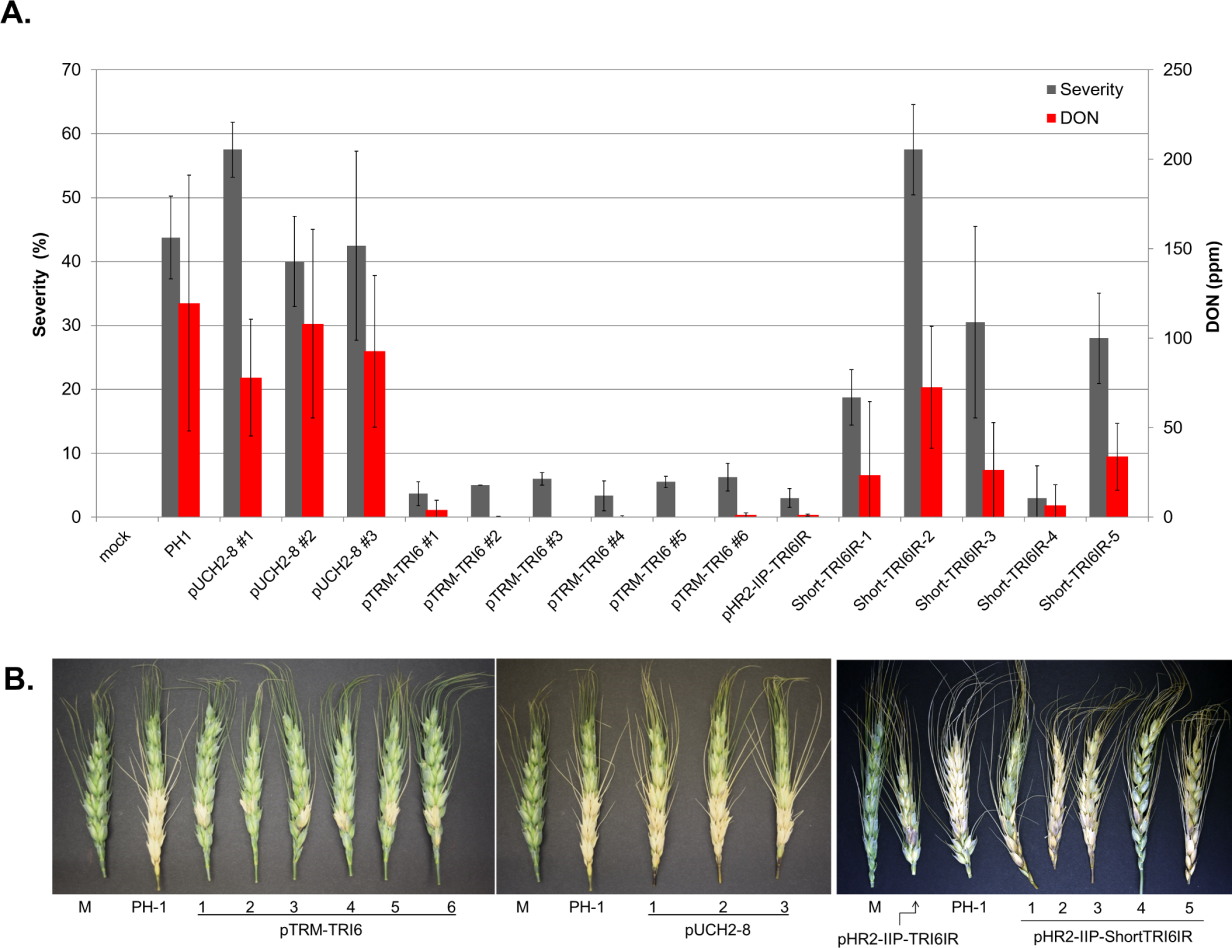
Virulence of RNAi mutant strains and PH1 point-inoculated on wheat cultivar Klasic. (A) Disease severity and DON content of infected kernals. (B) pTRM-TRI6 vs. PH1; pUCH2-8 vs. PH1; pHR2-IIP-TRI6IR and pHR2-IIP-shortTRI6IRs vs PH1 visual infections on wheat. Line bars denote standard errors of three experiments. (M = Mock inoculated).

Measurements of virulence and DON production on wheat spikes were done to verify the reduced virulence and DON accumulation phenotype reported by McDonald et al. (2005). Virulence on wheat was measured based on the spread of disease within the heads from a single inoculated floret, a process that is facilitated by DON, particularly in cultivars lacking the *Fhb1* locus [39] such as the cultivar Klasic that was used for these assays. For the ~600 bp RNAi mutant strains, FHB symptoms were confined primarily to the inoculated floret. In contrast, significant spread to flanking florets was observed for PH1 and pUCH2-8 mutant strains (Fig 2B). Wheat kernels infected with the ~600 bp RNAi *TRI6* mutant strains contained 100-fold less DON (average of pTRM-TRI6 0.96 μg g^-1^ for pTRM-TRI6 and 1.16 μg g^-1^ for pHR2-IIP-TRI6, pHR2-IIP-TRI6 was not tested) than PH1 (119.6 μg g^-1^) and pUCH2-8 mutant strains (average of 92.9 μg g^-1^). Similar phenotypes were observed for both random integration and integration via homologous recombination. Inoculation with mutant strains containing various short *TRI6* fragments resulted in greater severity and more DON than the ~600 bp TRI6 RNAi mutant strains (Fig 2A). DON concentrations ranged from 6.59 ± 5.00 μg g^-1^ (fragment 4) to 72.57 ± 7.07 μg g^-1^ (fragment 2).

Virulence and DON production was determined also on barley. Unlike most wheat cultivars (i.e. those without *Fhb1*), barley exhibits resistance to the spread of infection within the spike (Type II resistance, [40]), and DON is not known to influence this spread in barley. Therefore, virulence and mycotoxin production for pTRM-TRI6 RNAi mutant strains on barley were tested by dip-inoculating entire barley spikes into spore suspensions. An example of barley infection is shown in Fig K in S1 File. Infection severities (percentage of infected floret) averaged 80% for PH1 and pUCH2-8 mutant strains and 95% for the pTRM-TRI6 mutant strains 2–6 (Fig K in S1 File). DON accumulation in infected barley kernels was dramatically reduced (32-fold) for pTRM-TRI6 mutant strains compared to PH1 and pUCH2-8 (18 μg g^-1^ versus 694 μg g^-1^ and 570 μg g^-1^, respectively, Fig K in S1 File).

### 2.3 Small RNA profiles of pTRM-TRI6, full-length, and short fragment mutant strains

Rarefaction analysis of unique small RNA species mapping to *TRI6* indicated that 3 to 6 million reads would capture approximately 75% to 89% of all unique sRNA species (Fig L in S1 File). All data presented below was derived from assays that provided > 3 million reads with one exception (2.9 million reads).

**Fig 3:**
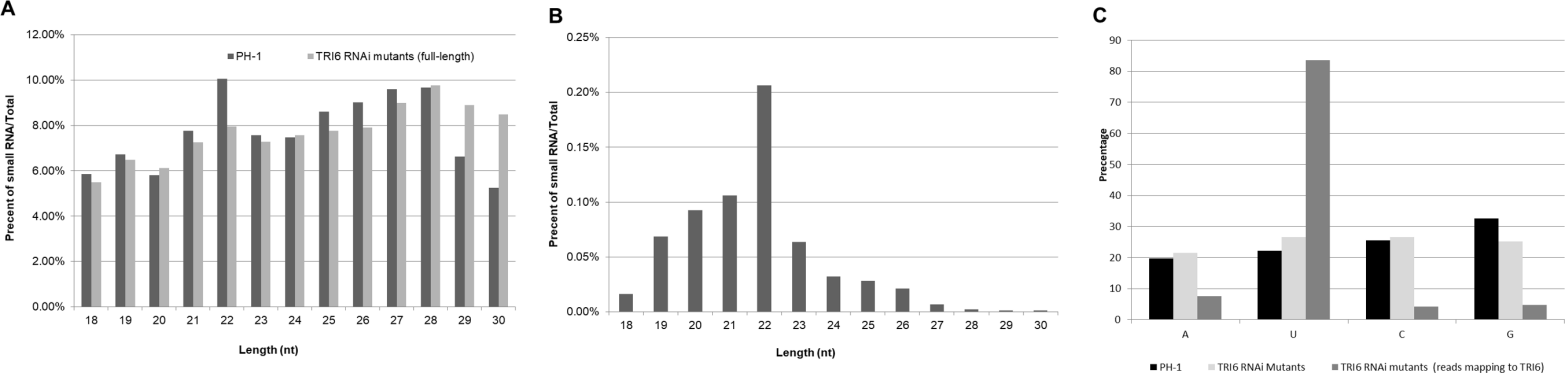
The percentage of various sRNAs by length (nt) for (A) total sRNAs and for (B) sRNAs that mapped to *TRI6*. (C) AUGC proportion of the first base in small RNA in *TRI6* RNAi mutants reads that map to *TRI6*.

Details of sRNA sequence results are outlined in Table 1 with individual mutant strains reported (technical reps were pooled for the analysis below). Most sRNA species were present in one or two copies. The 20 most-abundant species represented ~45% of the sRNA read mapping to *TRI6* (Table 2). The distributions of total background sRNAs (as a percent of total sRNAs) by length (18–30 nt) for PH1 and *TRI6* RNAi mutant strains is shown in Fig 3A; similar results have been reported in other studies of filamentous fungi [41, 42]. Interestingly, there was an elevation in 22mers in PH1 strains compared to*TRI6* RNAi mutant strains. Further analysis revealed that an increased accumulation of the top two 22mer species 5’–GGCCUGGCUGGCCGGUCCGCCU-3’ and 5’- CCGGGUGCUGAUGCCCUUGGCA- 3’ accounts for the majority of the difference (Fig M in S1 File). BLAST analysis identifies these species as ungrouped rRNAs and it is unclear if this is an artifact or an important observation that relates to mycotoxin synthesis under these conditions. It is worth noting that DON and related mycotoxins cause degradation of the 18S and 28S rRNA [43, 44] as part of the DON-induced translation inhibition of protein synthesis ([45] and references within).

**Table 2:** Small RNA sequencing details.

Strain	Tech. reps.	Total Reads	Total siRNAs Mapped to TRI6*	% reads mapped to TRI6*
**PH1**	1	3,067,468	271	0.01%
**pTRM-TRI6**				
#1	2	6,212,330	135,296	2.18%
#4	2	4,620,584	84,580	1.83%
#5	2	6,879,375	32,569	0.47%
**pHR1-IIP-TRI6IR**				
#1	1	2,990,497	11,012	0.37%
#2	1	3,779,238	1,473	0.04%
#3	1	3,290,804	4,661	0.14%
#4	1	4,103,946	17,186	0.42%
**pHR2-IIP-TRI6IR**				
#1	1	4,116,341	19,822	0.48%
#2	1	2,903,794	98,771	3.29%
#3	1	4,168,770	24,172	0.58%
#4	1	3,868,812	86,924	2.20%
**pHR1-IIP-TRI6IR-1**				
#1	2	9,675,922	8,622	0.09%
**pHR1-IIP-TRI6IR-2**				
#1	2	5,193,283	8,677	0.17%
**pHR1-IIP-TRI6IR-3**				
#1	2	3,507,432	2,150	0.10%
**pHR1-IIP-TRI6IR-4**				
#1	2	12,058,536	6,344	0.05%
**pHR1-IIP-TRI6IR-5**				
#1	2	11,883,688	13,592	0.11%

*Size range from 15–30 nt.

The sRNAs (assumed to be siRNAs) that mapped to *TRI6* in the *TRI6* RNAi mutant strains were mostly 22 nt or 21 nt long (Fig 3B) and the majority of siRNA had uracil at the 5' end (~83%) (Fig 3C). These characteristics are consistent with DICER-processing and ARGONAUTE’s affinity to bind siRNA [42, 46, 47, 48, 49, 50, 51]. To test whether dsRNA homologous to *TRI6* was subject to phased processing by DICER, the likelihood of siRNA starting at a particular position was analyzed using the calculation from [52]. No pattern of phased processing was discovered (Fig N in S1 File).

**Fig 4:**
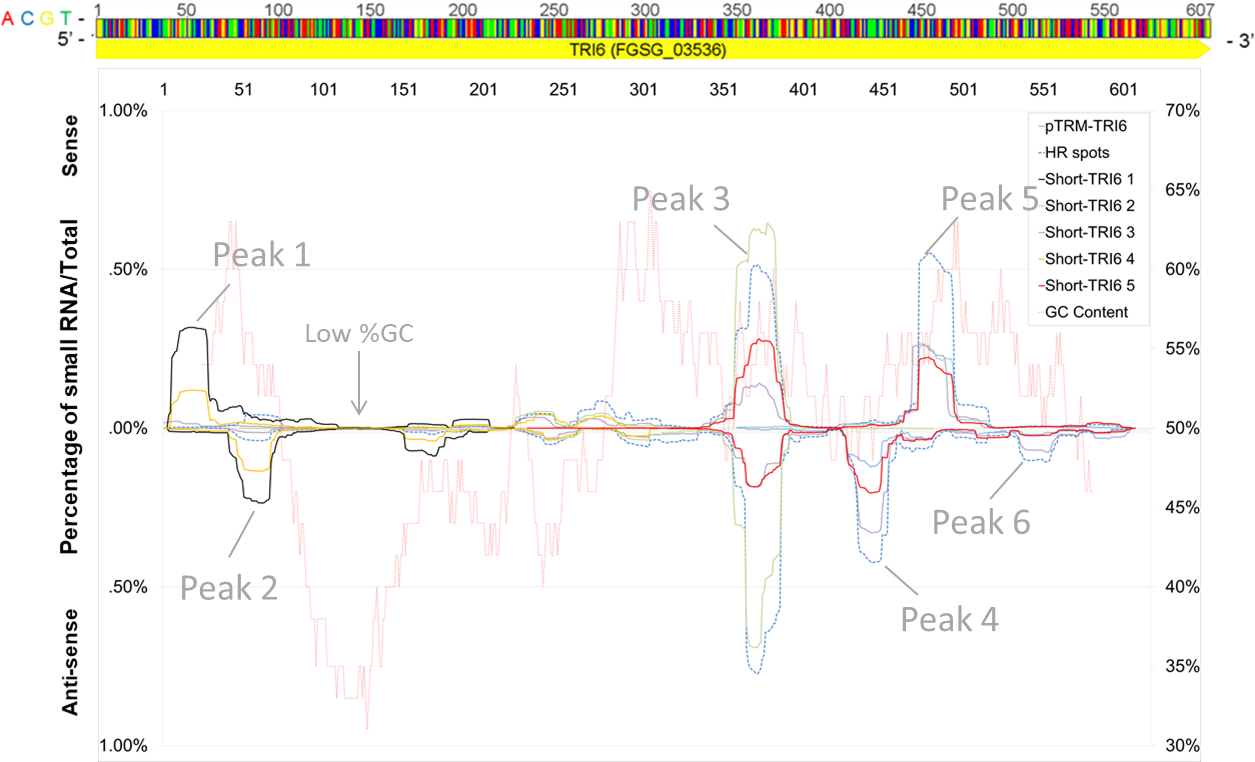
The average small RNA profiles (map position and relative abundance) of sRNA that mapped to *TRI6* (x axis is *TRI6* from 5’– 3’) from mutants containing either pTRM-TRI6, HR spots1 and 2 and the Short *TRI6* IRs (1–5).

A discontinuous distribution was revealed when all sRNAs that mapped to *TRI6* were plotted according their map position and relative abundance (Fig 4). Three major peaks (~72% of mapped sRNA) and three minor peaks (~10% of mapped sRNA) were noted (Table 3). The most abundant sRNAs mapped to these peaks (Table 4). Although peak positions were nearly identical among the mutant strains, apparent differences in their height (abundance) were noted (Fig 4). Interestingly, these peaks of abundant sRNA mapped to regions of *TRI6* that were > 47% in GC content (Fig 4). There was a notable lack of siRNA aligning to regions of *TRI6* with the lowest GC content (32%) (Fig 4). The replication of mapped sRNAs for each IR construct is shown in Fig O in S1 File. Generation of secondary sRNA upstream and downstream of the genomic region of *TRI6*, with introduction of the TRI6-IR transgene, was not observed. Additionally, the non-IR components of IR cassette -including the GDPA promoter, GFP fragment (loop), and the terminator- did not map sRNA (Data not shown).a

**Table 3:** The 20 most-abundant siRNA species that mapped to *TRI6* (from all full-length *TRI6* RNAi mutant strains analyzed).

#	Small interfering RNA	Length	Count	% of mapped reads	sense/anti-sense	5'	3'	Peak	%GC
1	UCGGCACCAAAGGCUUUGCGAC	22	34744	11.07	sense	473	494	5	59.1
2	UGACUGAGGGCAUUCUGAG	19	16723	5.33	anti-sense	435	453	4	52.6
3	UCCCGUCCGCUUUCAAAGACUG	22	15816	5.04	anti-sense	365	386	3	54.5
4	UGAGGGCAUUCUGAGUAGCGAC	22	14290	4.55	anti-sense	428	449	4	54.5
5	UGACUGAGGGCAUUCUGAGU	20	12440	3.96	anti-sense	434	453	4	50.0
6	UUUCAAAGACUGUGGCCGAC	20	6108	1.95	anti-sense	357	376	3	50.0
7	UGACUGAGGGCAUUCUGAGUA	21	5061	1.61	anti-sense	433	453	4	47.6
8	UCGGCACCAAAGGCUUUGCGA	21	4881	1.56	sense	473	493	5	57.1
9	UCGGCACCAAAGGCUUUGCGACU	23	4610	1.47	sense	473	495	5	56.5
10	UCGCAAGGACCGUGCUCGGCA	21	4379	1.40	sense	495	515	5	66.7
11	UCACUACGAAUCUUGGAGCGCCU	23	3652	1.16	sense	20	42	1	52.2
12	UCGGCCACAGUCUUUGAAAGCGGACG	26	3032	0.97	sense	358	383	3	57.7
13	UGAGCUGACUGAGGGCAUUCUGAG	24	2421	0.77	anti-sense	435	458	4	54.2
14	UCUUUGAAAGCGGACGGGACU	21	2412	0.77	sense	368	388	3	52.4
15	UUCCCUAGUUGCAAGUCGGCC	21	2293	0.73	sense	357	377	3	57.1
16	UGAGGGCAUUCUGAGUAGCGACAGA	25	2197	0.70	anti-sense	425	449	4	52.0
17	UGUUGUCCUUCCUUGUCUUGCC	22	2162	0.69	anti-sense	551	572	6	50.0
18	UUUCAAAGACUGUGGCCGACU	21	1929	0.61	anti-sense	370	390	3	47.6
19	UUGCCAAGGGCACCGCACUG	20	1788	0.57	anti-sense	536	555	6	65.0
20	UCUUUGAAAGCGGACGGGA	19	1778	0.57	sense	368	386	3	52.6

**Table 4:** Peaks associated with abundant siRNA production (from all full-length *TRI6* RNAi mutant strains analyzed).

Peak	Length	Count	% of mapped reads	sense/anti-sense	5'	3'	%GC
1	50	8120	2.11	sense	1	50	62.0
2	50	8916	2.31	**anti-sense**	51	100	52.0
3	50	95372	24.75	sense/**anti-sense**	361	410	56.0
4	50	92526	24.01	**anti-sense**	428	477	52.0
5	50	88688	23.02	sense	478	527	60.0
6	50	20219	5.25	**anti-sense**	542	591	52.0
Low GC%	50	77	0.02	-	107	156	34.0
*TRI6*	607	-	-	-	1	607	50.4

## Discussion

Introduction of RNAi vectors that targeted nearly the full length of *TRI6* into *F*. *graminearum* PH1 created strains with properties similar to strains reported by McDonald et al. (2005), as shown by the virulence and DON assays on wheat. The current study investigated the mechanisms behind these phenotypes and showed that *TRI5* expression (which is positively regulated by *TRI6*) was downregulated in all of these *TRI6* RNAi mutant strains, likely due to the presence of numerous siRNAs specific to *TRI6*. Although the expression of the *TRI6* as an inverted repeat prevented direct measurement of native *TRI6*, evidence of *TRI6* silencing included: 1) the intact, unperturbed nature of the native locus; 2) the presence of numerous siRNAs specific to *TRI6* in each mutant but not in the wildtype or control strains; and 3) the reduced expression of *TRI5*. The evidence that *TRI6-*specific sRNA are specifically siRNA includes preferential accumulation of 21 and 22 nt species and that the 5'- terminal base of the majority of siRNAs was uracil. Both of these phenomena are hallmarks of RNAi processing by the RISC system [42, 53, 54], and they are conserved across many animal, plant, and fungal species [55].

The central understanding of RNAi is that suppression of target genes is mediated by complementary siRNAs that are produced as a result of DICER-mediated processing of dsRNA. The exact siRNA sequence and concentration can affect the efficiency of target gene downregulation [16]. In the case of long dsRNAs, multiple species of siRNAs have been detected in filamentous fungi, but not in equal quantities. For instance, Ghag et al. (2014) showed difference of three orders of magnitude between the least and most abundant species of siRNA. The current study, and two other recent studies, provided additional insight by mapping siRNAs to the targeted sequence. These assays showed distinct preferences for siRNAs to map to specific regions of the dsRNA from which they were derived [30, 56]. Furthermore, the current study is the first to report the distribution of siRNAs among biological replicates (*e*.*g*., comparisons among mutant strains containing identical RNAi vectors), and to show that despite significant variability in the total number of *TRI6-*specific siRNAs (perhaps a result of genomic position effects), their distribution was nearly identical. Furthermore, variation in the number of *TRI6-*specific sRNAs was not correlated with silencing efficacy (compare data for pTRM-TRI6 mutant lines 1, 4;—Figs 1 and 4 and Table 2. However, this last point must be interpreted cautiously as these experiments are not sufficient to fully address this point, and it cannot be ruled out that this result may reflect an artifact of the RNA analysis process.

The siRNA profiles resulting from the short (250–350 bp) RNAi vectors were remarkably similar to those resulting from the longer RNAi vectors for regions targeted by the short vectors. This is a novel observation that suggests that specific sequences may generate specific sRNA populations regardless of their upstream and downstream contexts. A practical consequence may be that specific IR fragments may be considered as “modules” that could be expected to retain their silencing capacity when combined with other sequences, for instance in an IR construct designed to target multiple genes. Further investigation, including similar studies of other gene targets as well as investigating the effects of various combinations of IR fragments in RNAi vectors, is necessary to confirm or refute this observation.

The differences in silencing efficiencies achieved with the various RNAi constructs shows that not all regions are equally efficient at silencing *TRI6*. This may be related to specific siRNAs that are produced from a given region. The higher silencing efficiency of the long siRNA vector may be a result of several siRNAs, derived from multiple regions of the dsRNA homologous to *TRI6*, that have high silencing efficiencies (Reviewed in [57]). Given that the phased-processing effect was not measured in our siRNA productions, these results also suggest that neither phased processing by DICER not the starting position of processing significantly influence on the populations of siRNA. The results also are consistent with previous observations of preferential DICER processing of regions with higher GC content [30, 58, 59].

The discontinuous distribution profiles of siRNA mapping to their target seen in this study are interesting and have been reported in other studies [30, 60, 61], even when siRNA are derived from processing exogenously-supplied dsRNA (Koch et al., 2016). The present study shows, as does Koch et al. (2016), that the amount of sense siRNAs is not merely a reflection of antisense species, nor do they exhibit the preference towards the antisense sRNA duplexes that result from degradation of the passenger siRNA (also known as miRNA*) [62, 63, 64]. The mechanism(s) responsible for these observations are unclear. Preferential accumulation of specific siRNAs might result from amplification of specific regions of dsRNA by RNA-dependent RNA polymerase (RdRp), as found in arabidopsis [65]. *F*. *graminearum* has five predicted RdRp [66]. No evidence of RdRp-directed amplification beyond the *TRI6* sequences included in our construct were detected in this study. However, this study does not test directly for RdRp function. Other factors may play a role in guiding siRNA production, such as RNA secondary structure [67].

This research and siRNA observations demonstrate some factors governing the production of siRNA, and how processing patterns might affect rational design of RNAi vectors that will produce effective siRNAs. For instance, these results teach that random selection of sequences homologous to a specific target gene is unlikely to result in optimal silencing. Although inclusion of longer dsRNA sequences homologous to a target gene may result in optimal silencing with little optimization, targeting multiple genes would require relatively long vectors. Such vectors may be unwieldy to manipulate, and they will likely produce a greater number of siRNA species and thus increase the likelihood of off-target effects [68]. Efficient, minimal-length RNAi vectors targeting multiple genes are most likely to result from careful selection of target sequence based on empirical determinations.

## Supporting information

S1 File Plasmid construction and additional analyses. Supplemental information for primers used, plasmid construction, inoculation on barley, and supplemental information and analyses of Illumina sequencing data.
Table A. List of primers used in this study
Table B. Genetic characterization of the pTRM-TRI6 mutant strains used in this study
Procedure A. Supporting methods to identify and map random genomic integrations of pTRM-TRI6.
Fig A. Diagram of the RNAi plasmid pTRM-TRI6 and pUCH2-8 used for transformation and silencing the expression of *TRI6*.
Fig B. Assembled junction between plasmid and genomic DNA used to predict insertion site of randomly integrated pTRM-TRI6.
Fig C. Alignment of reads to pTRM-TRI6 sequence.
Fig D. Estimation of transgenic copy number using qPCR.
Fig E. PCR confirmation of site disruption based on assembly prediction of pTRM-TRI6 insertion.
Fig F. PCR indicating intact native *TRI6* in pTRM-TRI6 mutants with PCR.
Fig G. Construction of full-length inverted-repeat integration plasmid for directed genomic integration by homologous recombination.
Fig H. Synthesized DNA fragments for inverted repeat constructs.
Fig I. Construction of short inverted repeat vectors.
Fig J. DON production of mutants in rice culture media.
Fig K. Virulence of mutant strains pUCH2-8 and pTRM-TRI6 compared to PH1 on barley cultivar Golden Promise.
Fig L. Rarefaction analysis of unique small RNA species.
Fig M. Small RNA comparison of non-mapping 22mers from PH1 and all *TRI6* RNAi mutant strains tested.
Fig N. siRNA phased-processing calculations.
Fig O. Small RNA profiles of all mutants in this study.
(PDF)
