# Supplementary material for: Silencing efficiency of dsRNA fragments targeting Fusarium graminearum TRI6 and patterns of small interfering RNA associated with reduced virulence and mycotoxin production
Source: PLoS One. 2018 Aug 30;13(8):e0202798. doi: 10.1371/journal.pone.0202798 (PMC6116998; doi:10.1371/journal.pone.0202798)
Supplement: S1 File — Supplemental information for primers used, plasmid construction, inoculation on barley, and supplemental information and analyses of Illumina sequencing data. Table A. List of primers used in this study Table B. Genetic characterization of the pTRM-TRI6 mutant strains used in this study Procedure A. Supporting methods to identify and map random genomic integrations of pTRM-TRI6. Fig A. Diagram of the RNAi plasmid pTRM-TRI6 and pUCH2-8 used for transformation and silencing the expression of TRI6. Fig B. Assembled junction between plasmid and genomic DNA used to predict insertion site of randomly integrated pTRM-TRI6. Fig C. Alignment of reads to pTRM-TRI6 sequence. Fig D. Estimation of transgenic copy number using qPCR. Fig E. PCR confirmation of site disruption based on assembly prediction of pTRM-TRI6 insertion. Fig F. PCR indicating intact native TRI6 in pTRM-TRI6 mutants with PCR. Fig G. Construction of full-length inverted-repeat integration plasmid for directed genomic integration by homologous recombination. Fig H. Synthesized DNA fragments for inverted repeat constructs. Fig I. Construction of short inverted repeat vectors. Fig J. DON production of mutants in rice culture media. Fig K. Virulence of mutant strains pUCH2-8 and pTRM-TRI6 compared to PH1 on barley cultivar Golden Promise. Fig L. Rarefaction analysis of unique small RNA species. Fig M. Small RNA comparison of non-mapping 22mers from PH1 and all TRI6 RNAi mutant strains tested. Fig N. siRNA phased-processing calculations. Fig O. Small RNA profiles of all mutants in this study. (PDF) [file pone.0202798.s001.pdf]

Table A. List of primers used in this study

Primers used for creation of pHR1 + HR2 –IIP-TRI6IR

3' HR1 fw+gib	ACTCATCCTAGTATTATCTACTAACCCTTGATTCTCAAGTCCAACC
5' HR1 rv+gib	AAACACAAATAGAATTAAATCCCCTACGGTTCAGCCACTCAT

Creation of pHR2-IIP-short TRI6 IR vectors #1- #5
252 bp amplified GFP Fragment -

short-gfp frag fw	GAGGGCTATGTGCAGGAGAG
GFP-frag-rv	ATCCTCAATGTTGTGTCTGAT
Tri6 cDNA fw	GTACCAATGAGCTGCAGGGT
Tri6 cDNA rv	CGTAGTGAGATTCGGCCTCC
pHR2-IIP backbone	
IIP-GADPA_rv	TTAAACACTTACTTAGGGGAA
IIP-tTrpC-fw	GTACCGTTTAAACGATCCACT

Amplification of gene fragments

Tri6-1_assembly_p1	TCCCCTAAGTAAGTGTTTAATTGACTACCCTCGAAATGATTTACA
Tri6-1_assembly_p2	CTCTCCTGCACATAGCCCTCAGCAAACAAGTGGTTCTTCGGAGTA
Tri6-1_assembly_p3	TCAGACACAACATTGAGGATAGCAAACAAGTGGTTCTTCGGAGTA
Tri6-1_assembly_p4	GTGGATCGTTTAAACGGTACTTGACTACCCTCGAAATGATTTACA
Tri6-2_assembly_p1	TCCCCTAAGTAAGTGTTTAACCCACATACTCTCTACCAACGGTGG
Tri6-2_assembly_p2	CTCTCCTGCACATAGCCCTCGTTGCCGGTAATGCCGCCTAAAGTC
Tri6-2_assembly_p3	TCAGACACAACATTGAGGATGTTGCCGGTAATGCCGCCTAAAGTC
Tri6-2_assembly_p4	GTGGATCGTTTAAACGGTACCCCACATACTCTCTACCAACGGTGG
Tri6-3_assembly_p1	TCCCCTAAGTAAGTGTTTAAAAAGCGGACGGGACTTTAGGCGGCA
Tri6-3_assembly_p2	CTCTCCTGCACATAGCCCTCTCAACACTTATGTATCCGCCTATAG
Tri6-3_assembly_p3	TCAGACACAACATTGAGGATTCAACACTTATGTATCCGCCTATAG
Tri6-3_assembly_p4	GTGGATCGTTTAAACGGTACAAAGCGGACGGGACTTTAGGCGGCA
Tri6-4_assembly_p1	TCCCCTAAGTAAGTGTTTAATTGACTACCCTCGAAATGATTTACA
Tri6-4_assembly_p2	CTCTCCTGCACATAGCCCTCGGCGATAAGCTGGCTTGGCACTGCG
Tri6-4_assembly_p3	TCAGACACAACATTGAGGATGGCGATAAGCTGGCTTGGCACTGCG
Tri6-4_assembly_p4	GTGGATCGTTTAAACGGTACTTGACTACCCTCGAAATGATTTACA
Tri6-5_assembly_p1	TCCCCTAAGTAAGTGTTTAACCTTCACACGGCCAAGCAAATGCCC
Tri6-5_assembly_p2	CTCTCCTGCACATAGCCCTCTCAACACTTATGTATCCGCCTATAG
Tri6-5_assembly_p3	TCAGACACAACATTGAGGATTCAACACTTATGTATCCGCCTATAG
Tri6-5_assembly_p4	GTGGATCGTTTAAACGGTACCCTTCACACGGCCAAGCAAATGCCC

Other primers

gfp frag-qPCR fw	GAGCTGAAGGGCATTGACTT
gfp frag-qPCR rv	TGATGCCATTCTTTTGCTTG
Tri5QF (Seong et al., 2009)	TGAGGGATGTTGGATTGAGCAGTAC
Tri5QR (Seong et al., 2009)	TGCTTCCGCTCATCAAACAGGT
TubQF (Seong et al., 2009)	GTCAGTGCGGTAACCAAATCGT
TubQR (Seong et al., 2009)	CTCAGAGGTGCCGTTGTAAACACC

TRI6 5'flank fw	GACGCGTCTCGGATAAGAAT
TRI6 3'flank rv	TTTCGCAAATGTTGCCTTTT
pTRM-TRI6 GL #1 fw	CCTCCATCCTCTCTCCCACA
pTRM-TRI6 GL #1 rv	CGCGACGTAGCTGTACAGAT
pTRM-TRI6 GL #2 fw	CGCCACTGAGCAACATATGC
pTRM-TRI6 GL #2 rv	CGCCGTCAATAGAAAAGGCG
pTRM-TRI6 GL #3 fw	TTGCGCAAAGACAGACAACG
pTRM-TRI6 GL #3 rv	GCTGCGAACTATCAAAGCGG
pTRM-TRI6 GL #4 fw	TACAAGTAGCTACTCGCGCG
pTRM-TRI6 GL #4 rv	GTCAGTGTTTGCTTGGCCTG
pTRM-TRI6 GL #5 fw	AGCATTGCCCAGTCCTACAC
pTRM-TRI6 GL #5 rv	GGGAGATAGGCAGCGAGTTC
pTRM-TRI6 GL #6 fw	CAATGGCGCCGTACTTGATG
pTRM-TRI6 GL #6 rv	AAGACAAGGTGACTCGCAGG

Table B. Genetic characterization of the pTRM-TRI6 mutant strains used in this study

pTRM-TRI6 mutant strain	Copy number[a]	Intact pTRM-TRI6[b]	Predicted pTRM-TRI6 location[b]	pTRM-TRI6 location confirmation[c]	Intact TRI genes other than *TRI6*[b]	Intact native *TRI6*[c]	Putative Gene(s) disruption
# 1[d]	1	Yes	Chr 2 (4,798,558)	Yes	Intact	Intact	-
# 2	1	Yes	Unidentified*	No	Intact	Intact	-
# 3	1	Yes	Chr 1 (2,392,790)	Yes	Intact	Intact	FGSG_00729
# 4[d]	1	Yes	Chr 2 (6,802,059)	Yes	Intact	Intact	-
# 5[d]	1	Yes	Chr 2 (1,005,127)	Yes	Intact	Intact	-
# 6	2	Yes	1. Chr 1 (106,526)<br>2. Unidentified*	1.Yes<br>2.N/A	Intact	Intact	FGSG_00020<br>FGSG_00021

[a] Determined by qPCR

[b] Determined by NGS alignments to pTRM-TRI6 or PH1 sequence

[c] Determined by PCR across fungal genome/pTRM-TRI6 junction or across the native *TRI6* locus

[d] Strains used for small RNA sequencing

*Insertion too complex to identify genomic location by NGS (5x coverage).

Chr = Chromosome

Procedure A. Supporting methods to identify and map random genomic integrations of pTRM-TRI6

The location of the pTRM-TRI6 insertion into mutant strains was determined via whole genome sequencing that achieved 5x coverage of each strain. Data from the genetic characterization of these strains is shown in Table S2. The map location of the insertions was determined by analysis of genomic DNA sequences flanking the insertions (Fig B). Alignment of reads to the core pTRM-TRI6 plasmid sequence revealed fully intact copies of the plasmid in four of the six pTRM-TRI6 positive mutant strains. Two pTRM-TRI6 mutant strains, #1 and #2, had misalignments in the 5'-most region of the GDPA promoter (Fig B). Estimation of copy number via qPCR (Table B; Fig D) using primers specific to the green fluorescent protein (GFP) sequence linker fragment indicated single pTRM-TRI6 insertions in mutant strains 1 through 5. Two copies were detected in the pTRM-TRI6 mutant strain #6, presumably in different locations, but only one was determined by the sequence data. The locations predicted by alignment were confirmed for all strains, except #2, via PCR using primers specific to genomic DNA flanking the insertions. In this assay, the lack of PCR product is consistent with the presence of the insertion (Fig E). The band amplified from pTRM-TRI6 mutant strain #2 suggested the insertion of a partial copy of pTRM-TRI6 at the predicted site. Additionally, the insertions appeared to disrupt putative genes in pTRM-TRI6 mutant strains #3 and #6 (Table 1). Therefore, only mutant strains #1, 4, and 5 were included in the sRNA analysis.

The integrity of native TRI genes was investigated by alignment of sequence reads to wild type PH1 genomic sequence (www.ensemble.org; King et al. 2015) and by

PCR. Sequence analysis of all TRI genes other than *TRI6* showed them to be intact in all mutant strains (data not shown). Because these analyses could not distinguish between the native and introduced *TRI6* sequences, misalignments near the 5' and 3' ends were expected and observed in the mutant strains (Fig F). Therefore, a PCR test was conducted with genomic primers flanking *TRI6* to determine whether the native gene had been interrupted by homologous recombination with pTRM-TRI6 (as observed by Scherm et al., 2011). Products of the expected length were observed (Fig F; Table B), providing evidence that the native *TRI6* was intact.

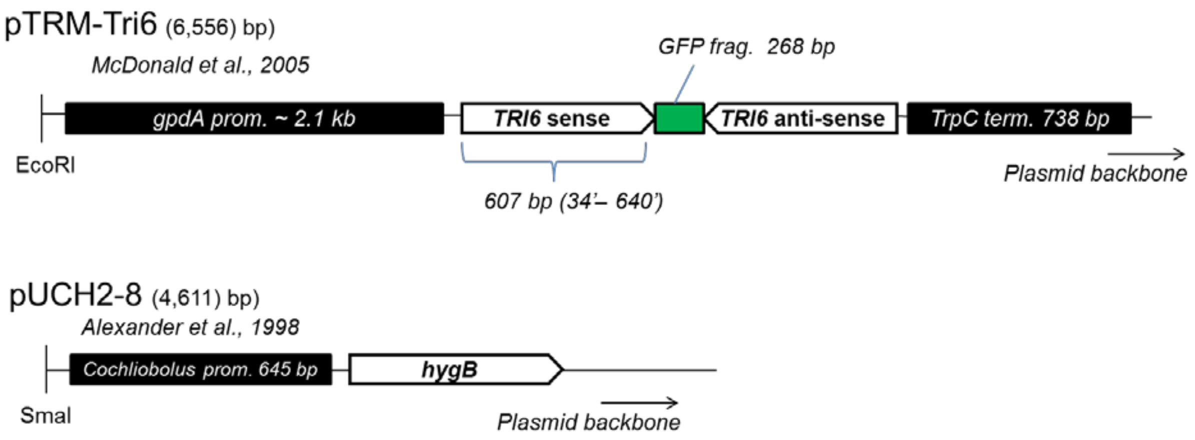

Fig A. Diagram of the RNAi plasmid pTRM-TRI6 and pUCH2-8 used for transformation and silencing the expression of *TRI6*. The pUCH2-8 plasmid containing hygB resistance gene was used for selecting transformants *F. graminearum* on hygromycin.

pTRM-TRI6 #1
TCACGAGGCCTTCCGTCGGCTCAGTATTTCAACTTCTAATGTAGAGTTCCTATGGC
ATCCTGCTTGGTCTCTACATTGGTAGTAGTTATGATACTGAACCGGCGAAGGGCAC
GCTCCCCGGCCTAAGGTTTGTAACAAATAGACATTACTCGCCGTCCTTATCATGGC
GACCACACCCGTCCTGTGGATCTGCACCCAATCGGCAGGCACGGGCGGCGATCT
CCAATCTGCGGGATCAGTCAGATCACCCGAGTGCGTGGGCATGACAATCGTGCCC
TGGGGACCAACACAATCCAGAAGGGCCTGAATCACTGCGACCGGCCCTCCCGCG
ACCCAGCCGAGCGAGCTTAGCGAACTGTG

pTRM-TRI6 #2
CCTACATACCTCGCTCTGCTAATCCTGTTACCAGTGGCTGCTGCCAGTGGCGATAA
GTCGTGTCTTACCGGGTTGGACTCAAGACGATAGTTACCGGATAAGGCGCAGCGG
TCGGGCTGAACGGGGGGTTCGTGCACACAGCCCAGCTTGGTGGCCGTTTCCAAC
ACTCGCAATCGCTACAAGAGTACGAGGCCGCTGTACCAGTACATTCCCCTGCGCC
CAGCGGCCCCCACCCAGACCGTCAACGGTTTCGCTCATTGGTCGTGGCGCTCACT
GCTGACGCGTAAACTGCCATCGTAG

pTRM-TRI6 #3
TTTCATTGTGCTCTGGGCGGACAACTCCATTGGAGGGCCACTTGAACGGGTGTGT
TACAGCCAATCAATTGGCGGTTGACCACTAAGAACACGCGCCTCTGCAGCTCTTCC
CTTTTTTACCCTTAATTTTTCTCGCGTAGGAAGGCGGGCGCTGATTTTARTTKTTKT
TTTSSTGSTTTTGCTGGTATTGACTTCAAATGAAGCTATACGCAGACGGTTTGATGG
GGGCTGAACGGCGGATAGACGCGTTTTAATCTGTAGTGGGCGCGATTCGCTGTTT
TGCTG

pTRM-TRI6 #4
GTCTCGAAGTATTGCTCTGTTAATTCTATGGAAGCATGCATCCGTAGTAATGTCAAT
CCATGCGTAACCAACATGGCGATCTACTCTAAAGTACCCAGTGACCAACATCATCA
CCAGTAAGGACGCACTCACATGCAACCAACGGTTGGATTCGGGACTGAACACGG
CCAAAAAATCATACTACACGATTCAAGTTTGAAACTATCTTTCTGTCAACTGATTAAT
GCTATTAATTGACAGGTACCCTGTCCCAAAGCTATTGGCGGGATATTCTGTTTGCA
GTTGGCTGACTTGAAGTAATCTCTGCAGATCTTTCGACACTGAAATACGTCGAGCC
TGCTCCGCTTG

pTRM-TRI6 #5
TGTGGCGCCAACTTTCGTCATGGTGCTGTCTGGCACGTTGCCTGATGCTTCACGCT
CAGAGGCTGGAGTCGTTGGGCCTCTGTGATGAGCCACGCAAGCCGTCATTACGCA
TTTTGTGTTCCTCGAGTAGCGAGGCAGTGTGCAGCGTTATTGGGCAATGCTGATGT
TATCTCTACACACAGGCTCAAATCAATAAGAAGAACGGTTCGTCTTTTTCGTTTATA
TCTTGCATCGTCCCAAAGCTATTGGCGGGATATTCTGTTTGCAGTT

pTRM-TRI6 #6
GCTGTAACGTTCAGTGATGATCTCTACCAAGATTGGAAATCAGGCGTCTGGTAGTA
AGCTGATAGAATAATGGCTGTCACAAGAAACATGCCATGAAAGTCGGTGACTCCTA
CTACTATGGGGCTGGCTGAACTTTATGTATAGATCCTATTGGCTGGCTGATATGGG
GGGCTTTCTAGGCCCCGTTGGATTCAATAGTGCGTTTGTACCCCGCGTCTTTTTCG
TTTATATCTTGCNATCGNNTCCCAAAGCTATTGGCGGGATATTCTGTTTGCAGTTGG
CTGA

Fig B. Assembled junction between plasmid and genomic DNA used to predict insertion site of randomly integrated pTRM-TRI6. *Fusarium graminearum* genomic sequence is represented in red.

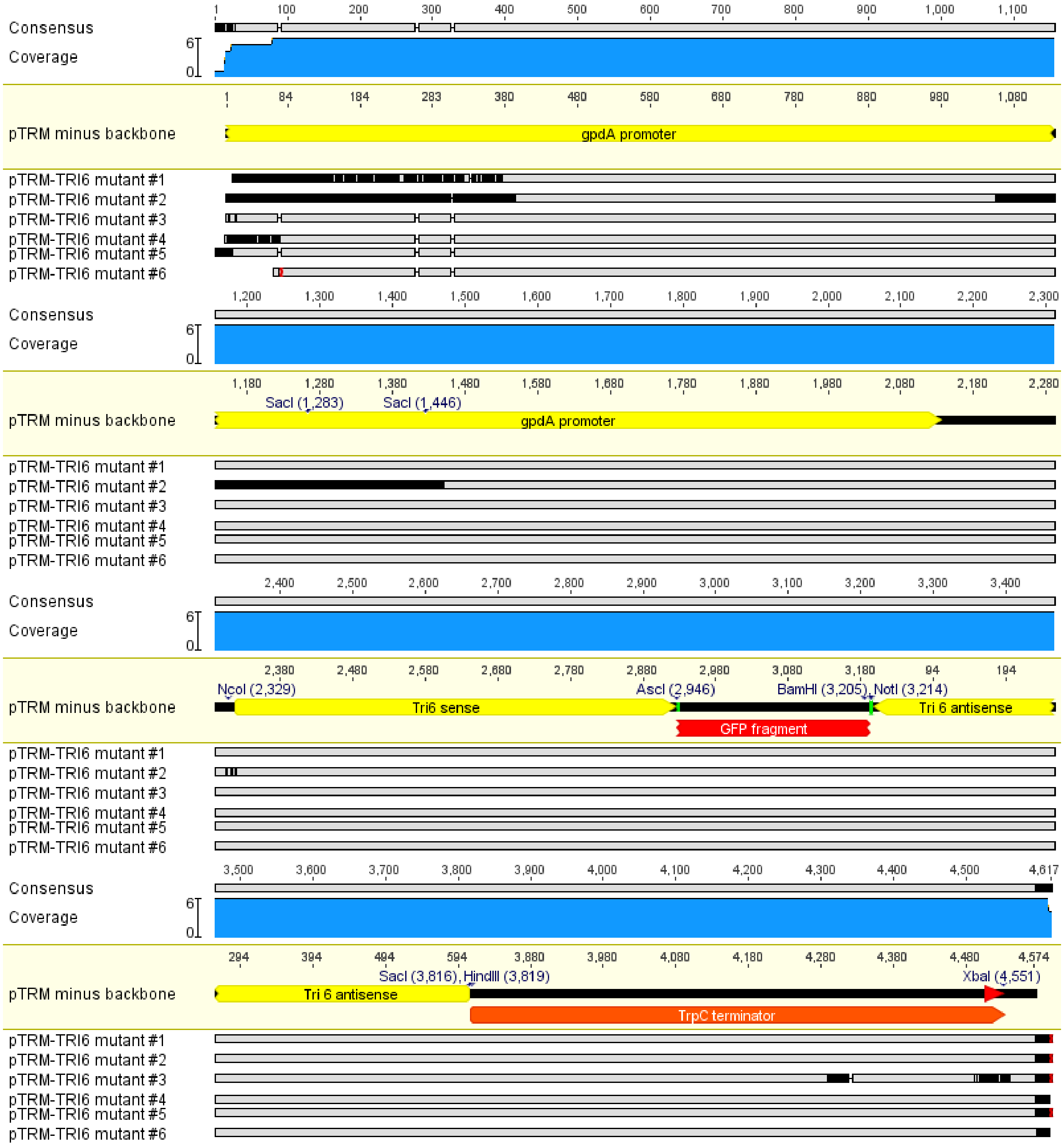

Fig C. Alignment of reads to pTRM-TRI6 sequence. This figure shows how intact the promotor, inverted repeat, and terminator are in the fungal genome for each transformant.

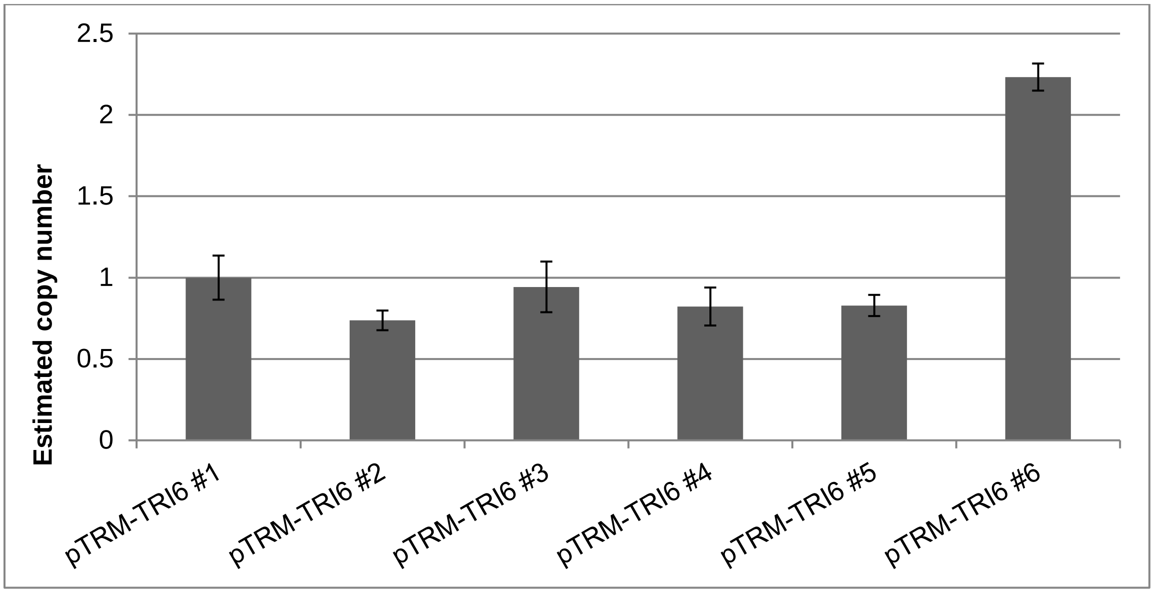

Fig D. Estimation of transgenic copy number using qPCR. Primers gfp frag-qPCR fw and gfp frag-qPCR rv amplifying the GFP fragment between the inverted repeat in the pTRM-TRI6 plasmid. Primers TubQF and TubQR were used to amplify β tubulin as a single copy reference gene. Line bars denote standard errors of one experiment with three technical replicates.

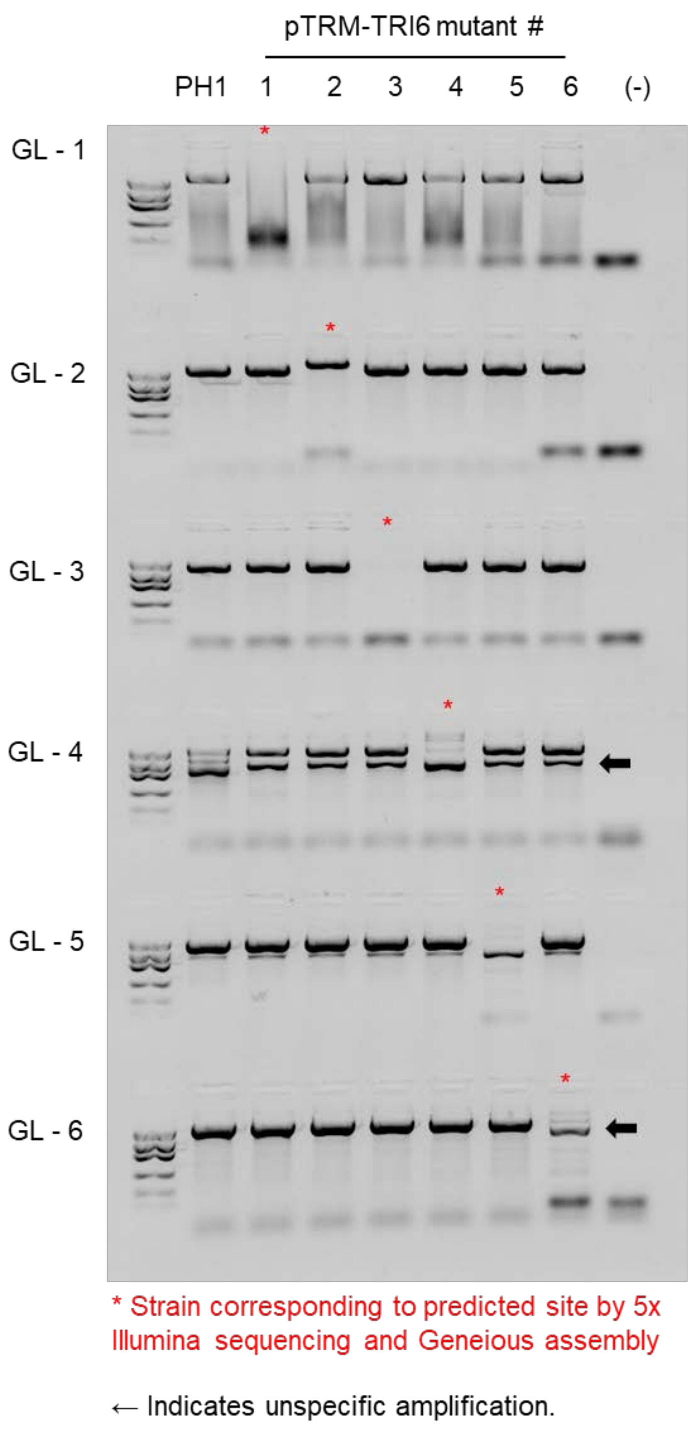

Fig E. PCR confirmation of site disruption based on assembly prediction of pTRM-TRI6 insertion. (GL) genomic location PCR used the primers in Table A.

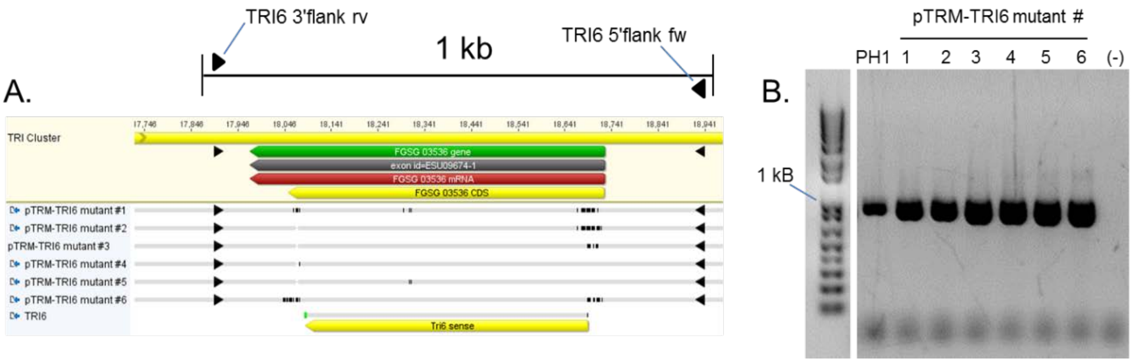

Fig

F. PCR indicating intact native TRI6 in pTRM-TRI6 mutants with PCR. Integrity of native TRI6 (A) consensus sequence from assembled NGS reads and misalignments at the junction of pTRM-TR6 IR in black. (B) PCR indicating intact native TRI6 with PCR spanning misalignments from NGS assembly

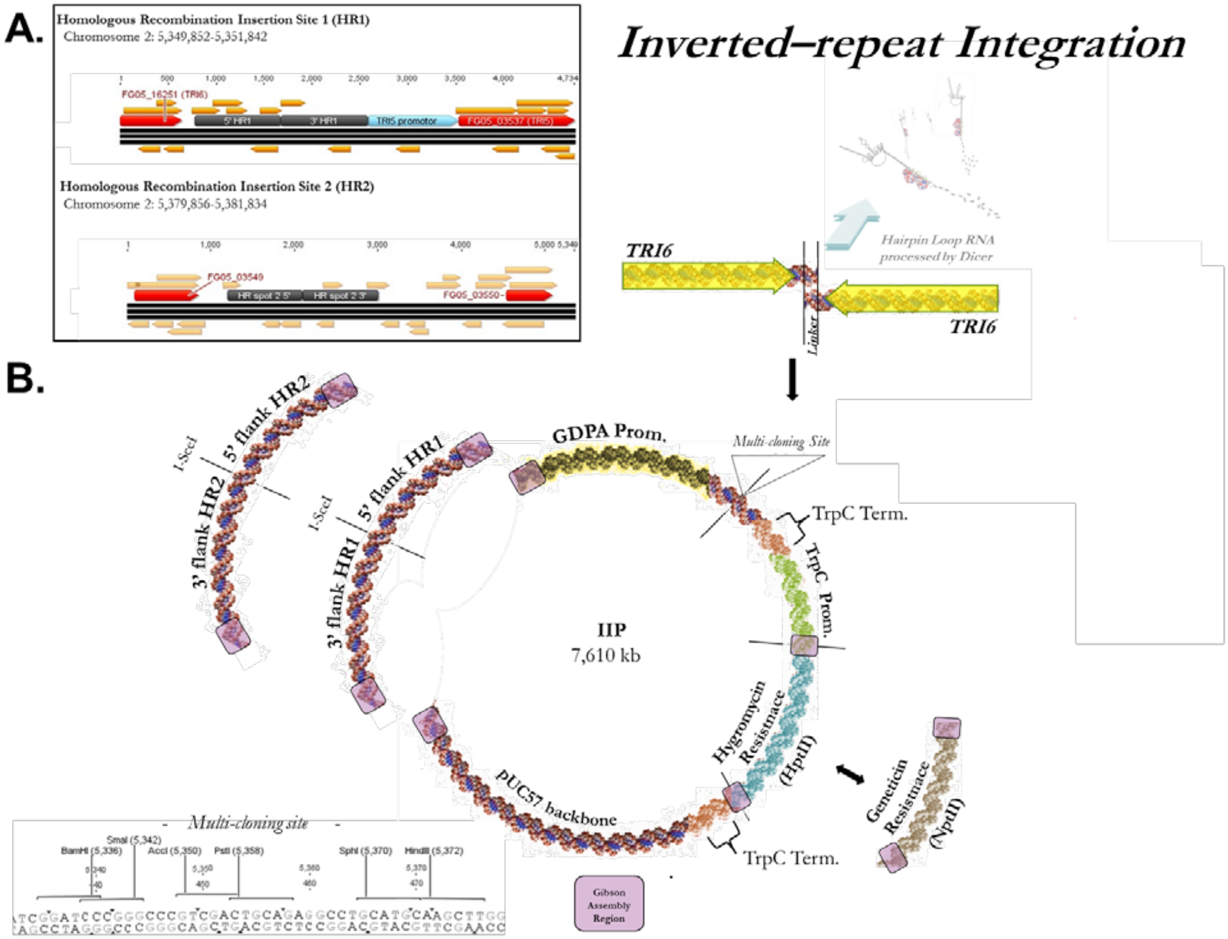

Fig G. Construction of full-length inverted-repeat integration plasmid for directed genomic integration by homologous recombination. (**A**) These sites were chosen to avoid all known and predicted genes. HR1 inserts transgenes between *TRI6* and *TRI5* in the *TRI* cluster. HR2 inserts transgenes between to genes just outside the TRI cluster. (**B**) IIP is designed to incorporate genes and RNAi inverted repeats by Gibson assembly or restriction cloning. Homologous recombination flanks are separated by I-SceI (fungal genome non-cutter).

Methods: The pHR2-IIP backbone was synthesized by Genscript (Scotch Plains, NJ) and includes a hygromycin resistance cassette and a gdpA promoter::sequence

insertion site::trypC terminator cassette (Fig G) plus sequences homologous to HR2 (Fig H). To create the pHR1-IIP backbone (for insertion into HR1), the pHR2-IIP backbone was PCR-amplified with primers 5' HR1 rv+gib and 3' HR1 fw+gib (Table S1). Next, 5’ and 3’ flanking homologous regions joined by an I-SceI restriction site (Fig H) were designed by gBlock (IDTDNA, Coralville, Iowa) and assembled using NEBuilder HiFi DNA Assembly master mix (New England Biolab Beverly, MA) HR1 . For pHR1-IIP-TRI6IR and pHR2-IIP-TRI6IR, 607 bp fragments of *TRI6* separated by a 252 bp *GFP* fragment were synthesized as a whole cassette (Genscript) and assembled into both pHR1-IIP and pHR2-IIP with restriction ligation (AscI/SpeI).

HR1 sequence IDT gBlock

ACTCATCCTAGTATTATCTACTAACCCTTGATTCTCAAGTCCAACCCTAATAGTGCC
CGGCGGAATGAGACGTTTTCGGGTGTCTGTAGCCGAGATGTGGATAGTAACGGTA
ACCCTAGTCAAATGAGACGTGGGCAGGGTTCATGGTTGTTGAACCTTGTTCATCAG
AATGTTGATGCCGTTGACCTACGGAATACCATCTTTCACATGTATTTGTTCCCAACC
CACGTGGCTATACCAACATCCGCTGTATATTCATTGGTTGGCTTTATATATTGATAC
AGGTAATTTCAAGATCGCGAGCGGGAATTTCCTTGTCGGATCAGAGAGATGCTCTA
CGCCGTGGGGGTAAAAGTCAAATCAGATAAGAGCACAGCAACCGGCAATGCTCAC
GGGCTACAGTGAATGTTCGTGATATGATTATGGCTTCCTCTATATCACTCACATATC
CACAGTTATTCCAAACAGTTCGAGTTCTGTAGATAGTGGATACGTCTTCCAGGATG
TATGTAATCTAGCAGCCGGTAGTTGAAACACCTGCCATGTAGAGGTGAGGAGCCC
AGCATCGCCAGTATGCACGAAGTACGGATATGATGATCTCAATTGTGCTTCCCCTG
CTGCTAGTGCGACCGGACTTGTCACATTTGCTGTATTCCCAAATTATGTCAGTTTCA
GTCAGCGATGACAGGCATGCCAGATAACCGTTTTCGTCATCATCGCCGTTAGATG
GACAAGGGCAGCGTAGCTTCAAACTGTGAAAACGGTTTCATGCATAATGCATGTGT
AGATTCCAATGGCGTGAACACCCCTACCGTGAACATCCTGATATCTTCGCATAGAT
AAGGATATGTCAAGACGGCAGACAGCGGCTATACGGACCATGTCCTAGAACTAAG
ACATGGGTTAATTGAAAACGGGATTCGGGATTCTGTACTCTTAGGGATAACAGGGT
AATGTATATCGATGCTGTTTCCAGCACCAATTCATAATATACTCCATAGTAGCCGAG
ACCCTGCAGCTCATTGGTACCAGCCCTGTTTTTATTGCACAATTAATGACACTCTAT
TCTGCACGCGACTCAACGGCTTTCAACACTTACATATTGCCTCTACGCGTGGCTGT
GTCTAAGCGAGATCAGCGCGGTCATTGTTATTGCTGCGTTGCTTTGGCCCCGTAC
GTTCCACAACAATCTGGCCACTGCAAGGTATTTGCGGCCGCTGGTTAAATTCTCTT
AGTAGTCTGCCTCATTTGGCAGGTGCCAATAATATATTTTACCCGCGTTTGGCTGG
CCATGCTACCCAAAAGGCTTCATACATATCTGGCCCATATCGTTTACTCGGTTCCA
CGTTTACGAGCATACGCCTCTCGTATCAATGTCGCAAACAAACATACGTCATTGGT
TACTATGCCGGGTTTCCGCGAACCATTGCTGTGCCGCAAAAGGATGCATCGGCAC
TCTAAAGATTGCGATCCTGTGTTAGAGGGTGACTTGAGTCAAGAAGTAACGTAACA
AGTGAGATAATGAAATTAATTACCTGAGGGCAATTTAAGGTTTCAACCTCCGAGGA
AAGCCATTCGTCGTGGGATATAATCTGCAATAGGGTAGGCTTTGCTGTTTTTTTCG
GAGCATTTGTGACTGTTTTGGATTGTGTCTTTGGGGTCCTTTTGCTTTCAAGGCTG
GTGAGTTTGCAAGGAACGAGTCAGTCACGGCAGGCACTGAGTCAAACACTGTTTC
GTAATAATCATGATTTGTTGTTGCAAAATTCCAACGAAGCTTTGCGCGGTCAGTCAA
ATTCGCACACGTCGATACTTTTTGCTGGCATTGGCCCAGTATTCATCCATGAGTGG
CTGAACCGTAGGGGATTTAATTCTATTTGTGTTTGATCGAGACC

I-SceI site

TRI6 IR with GFP fragment loop (Genscript)

GGCGCGCCGGGTCTCACTACGAATCTTGGAGCGCCTTGCCCCTCTTTGATCGAGT
TGCGTCTCCCGATCCTGCCAAGGACTTTGTCCCAGATCTAAACGACTATGAATCAC
CAACATTCGAAATAGATCTTCTCTCAGAAACTTATGACTTTGACAACTTCCCCACAT
ACTCTCTACCAACGGTGGATTCAACCAAGACTTTGTACTCCGAAGAACCACTTGTT
TGCTTCGACTTTGACTTCGCGAACCCGGCTATCGAAAATTATATAACCACATCGTC
GGGACTGTTGGACGCAGTGCCAAGCCAGCTTATCGCCCTTCCCACCTTCACACGG
CCAAGCAAATGCCCATTCCCTAGTTGCAAGTCGGCCACAGTCTTTGAAAGCGGAC
GGGACTTTAGGCGGCATTACCGGCAACACTTCAAGCGCTTTTTCTGTCGCTACTCA
GAATGCCCTCAGTCAGCTCAAGACCTGCAAGAAGTCGGCACCAAAGGCTTTGCGA
CTCGCAAGGACCGTGCTCGGCATGAGTCTAAGCACAAACCAACAGTGCGGTGCCC
TTGGCAAGACAAGGAAGGACAACAATGTCTGAGGGTCTTTAGCAGGGTGGATAAC
ATGGGAGGGCTATGTGCAGGAGAGAACCATCTTTTTCAAAGATGACGGGAACTAC
AAGACCCGCGCTGAAGTCAAGTTCGAAGGTGACACCCTGGTGAATAGAATCGAGC
TGAAGGGCATTGACTTTAAGGAGGATGGAAACATTCTCGGCCACAAGCTGGAATA
CAACTATAACTCCCACAATGTGTACATCATGGCCGACAAGCAAAAGAATGGCATCA
AGGTCAACTTCAAGATCAGACACAACATTGAGGATGGCCGCTAGTGATCTCGCATG
TTATCCACCCTGCTAAAGACCCTCAGACATTGTTGTCCTTCCTTGTCTTGCCAAGG
GCACCGCACTGTTGGTTTGTGCTTAGACTCATGCCGAGCACGGTCCTTGCGAGTC
GCAAAGCCTTTGGTGCCGACTTCTTGCAGGTCTTGAGCTGACTGAGGGCATTCTG
AGTAGCGACAGAAAAAGCGCTTGAAGTGTTGCCGGTAATGCCGCCTAAAGTCCCG
TCCGCTTTCAAAGACTGTGGCCGACTTGCAACTAGGGAATGGGCATTTGCTTGGC
CGTGTGAAGGTGGGAAGGGCGATAAGCTGGCTTGGCACTGCGTCCAACAGTCCC
GACGATGTGGTTATATAATTTTCGATAGCCGGGTTCGCGAAGTCAAAGTCGAAGCA
AACAAGTGGTTCTTCGGAGTACAAAGTCTTGGTTGAATCCACCGTTGGTAGAGAGT
ATGTGGGGAAGTTGTCAAAGTCATAAGTTTCTGAGAGAAGATCTATTTCGAATGTT
GGTGATTCATAGTCGTTTAGATCTGGGACAAAGTCCTTGGCAGGATCGGGAGACG
CAACTCGATCAAAGAGGGGCAAGGAGCTCACTAGTG

GFP fragment (loop)

AscI/SpeI

Fig H. Synthesized DNA fragments for inverted repeat constructs. Synthesized DNA fragments HR1 (IDT) and TRI6-IR with GFP fragment (Genscript) used for creation of RNAi vectors in this study

A.

B.

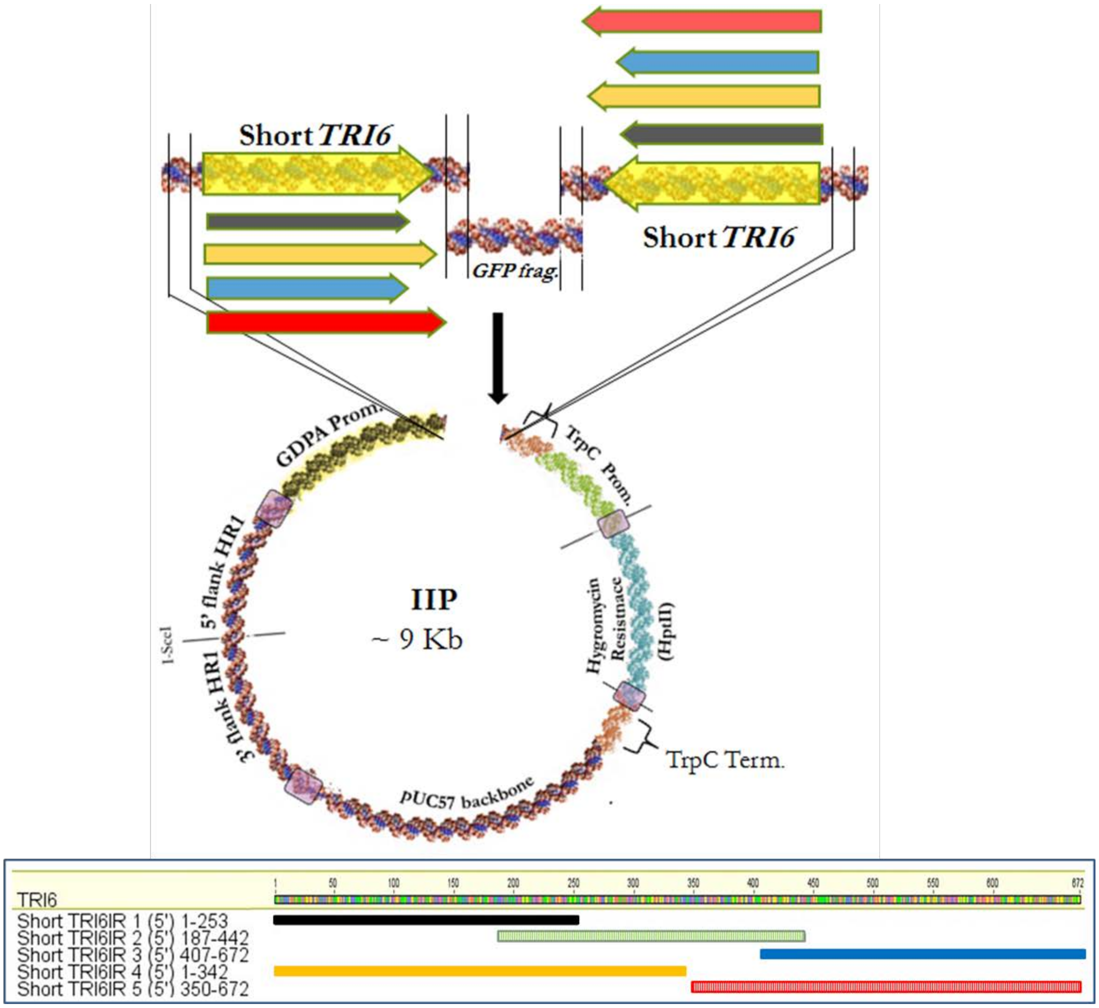

Fig I. Construction of short inverted repeat vectors. pHR2-IIP-ShortTRI6 fragments 1 - 5 (A) was created by Gibson Assembly with 4 primers (45 -mer) amplifyiing the sense and antisense orientation with the 252 bp GFP fragment and plasmid backbone (IIP + HygR). (B) Three ~ 250 bp regions and two ~350 bp regions spanning TRI6 (fragments 1 to 5) assembled into the RNAi vector are shown.

Methods: All primers for these assemblies are listed in Table S1. The *TRI6* fragments were individually amplified from a *TRI6* cDNA fragment with primers that added extensions to create a 20 bp overlaps with the gdpA promoter, the trpC terminator, the 252 bp GFP fragment (amplified via PCR), and HR2-IIP backbone (amplified via PCR)

(Table S1). Final assembly of all fragments were done with the NEBuilder HiFi DNA Assembly master mix (New England Biolabs, Ipswich, MA). The pHR2-IIP-ShortTRI6IR plasmids (1 to 5) are described in Fig I.

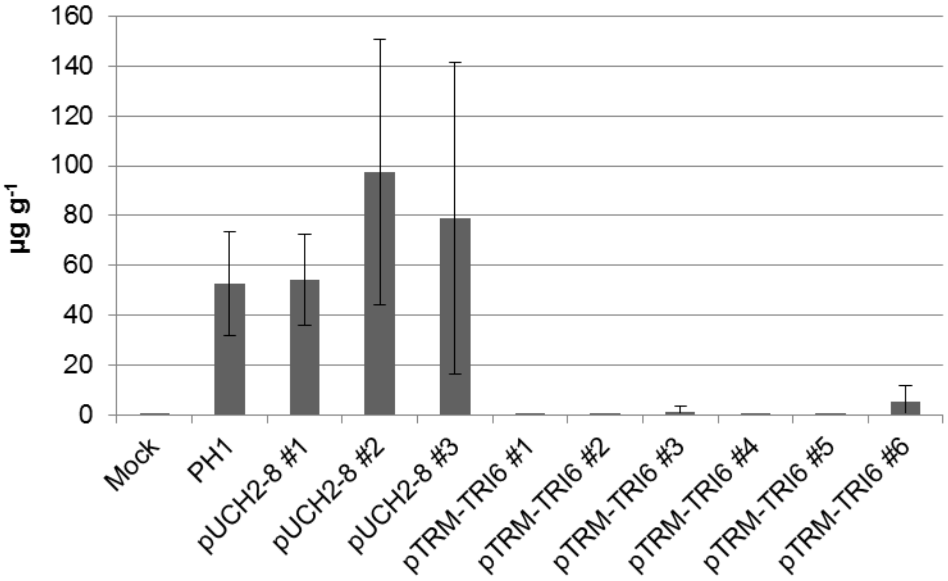

Fig J. DON production of mutants in rice culture media. Line bars denote standard errors of three experiments.

Methods: DON production in rice culture media was evaluated for each strain in three experiments, each with four technical reps (individual cultures). Rice culture media was prepared in individual scintillation vials by adding 5 g of dried, processed rice (food grade), rehydrating in 3 mL of water for 1 h, followed by autoclaving for 20 minutes (modified from Geraldo et al., 2006). Rice culture media was inoculated with 10 µl of $1x10^5$ conidia. Inoculated rice culture media was incubated at 25 °C for two weeks. Samples were freeze dried and pulverized using a SPEX freezer mill as described in the materials and methods section of the main paper.

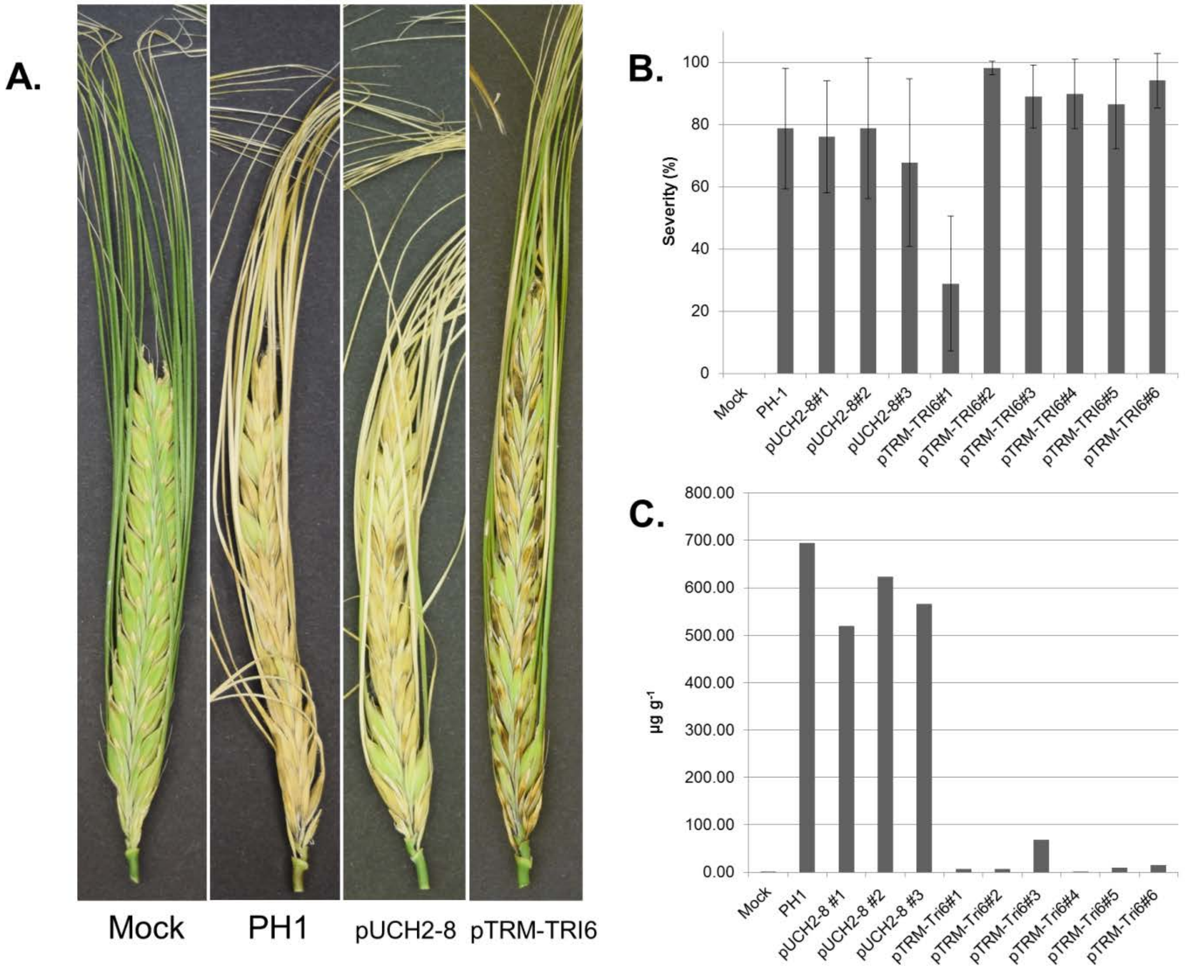

Fig K. Virulence of mutant strains pUCH2-8 and pTRM-TRI6 compared to PH1 on barley cultivar Golden Promise. (A) Representative infected spikes. (B) Disease severity. Line bars denote standard errors of one experiment (C) DON content of infected barley florets..

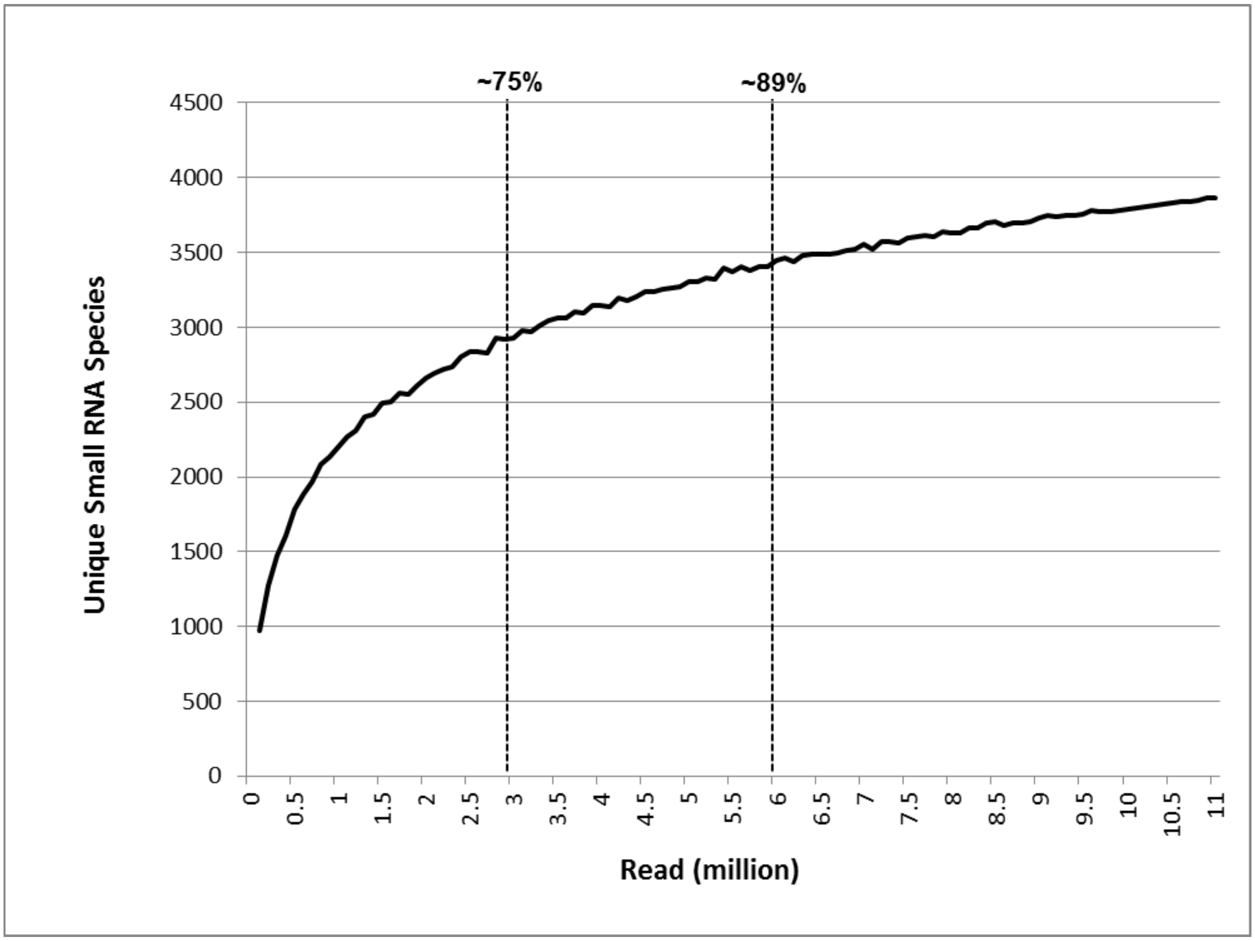

Fig L. Rarefaction analysis of unique small RNA species. Rarefaction analysis of unique small RNA species mapping to *TRI6* as a measurement of read depth, measuring from 11 million reads. Targeting 3 million and 6 million reads captures ~75% and 89%, respectively, of all unique small RNA species from 11 million reads. Increasing reads from 10 million to 11 million only captured 20 additional unique small RNAs mapping to *TRI6*.

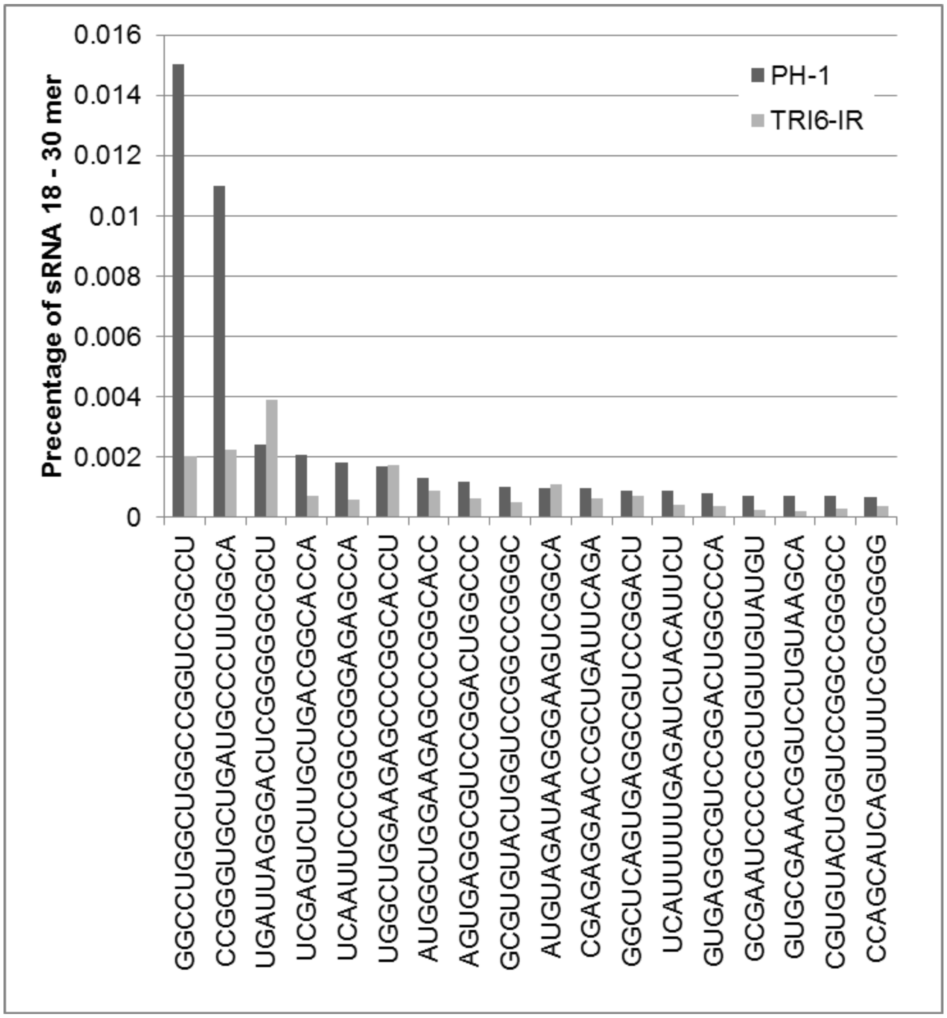

Fig M. Small RNA comparison of non-mapping 22mers from PH1 and all *TRI6* RNAi mutant strains tested. sRNA comparison of non-mapping 22mers from PH-1 and all *TRI6* RNAi mutant strains tested. In TBI media (3 dpi) revealed an increase in two species of 0.013016% and 0.008743% (of all sRNA 18-30 mer), respectively.

Phasing analysis. The frequency of distances separating all 23-mer 5′-end pairs (i, j) mapping to the same DNA strand was calculated using the following equation:

$$Frequency_D = \sum_{i,j} (Reads_i \cdot Reads_j)_D$$

where D = distance between sRNA 5′ ends

The frequency of distances separating pairs of 23-mer 5′ ends mapping to opposite strands of DNA was calculated separately using the same equation.

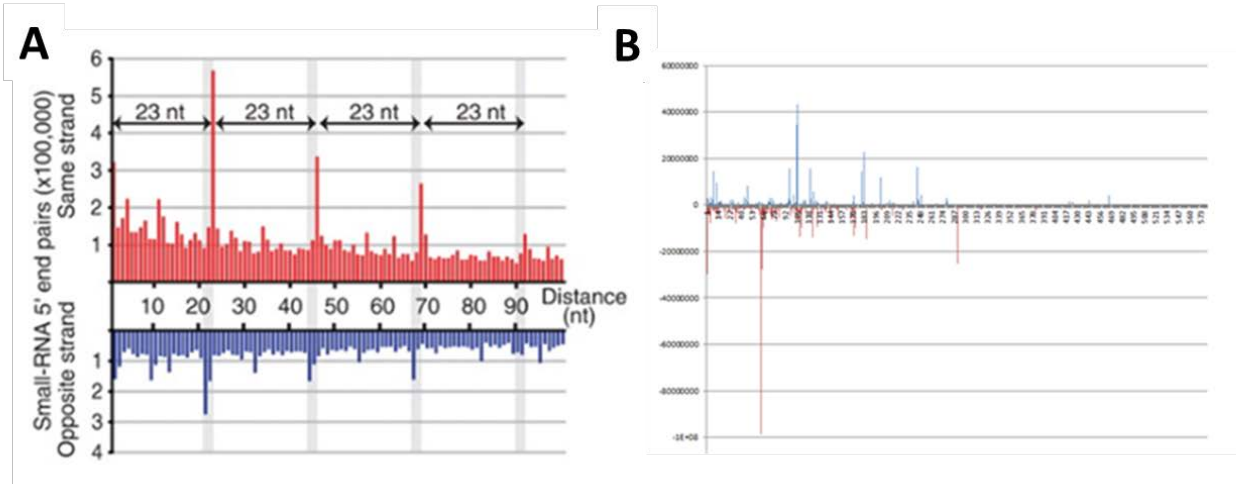

Fig N. siRNA phased-processing calculations. (A) Results from Drinnenberg et al., 2009 showing peaks every 23 nt, as an indication of phased processing. In comparison, the same calculation applied to 22nt from pTRM-TRI6 RNAi containing mutants (B) showed no discernable processing patterns.

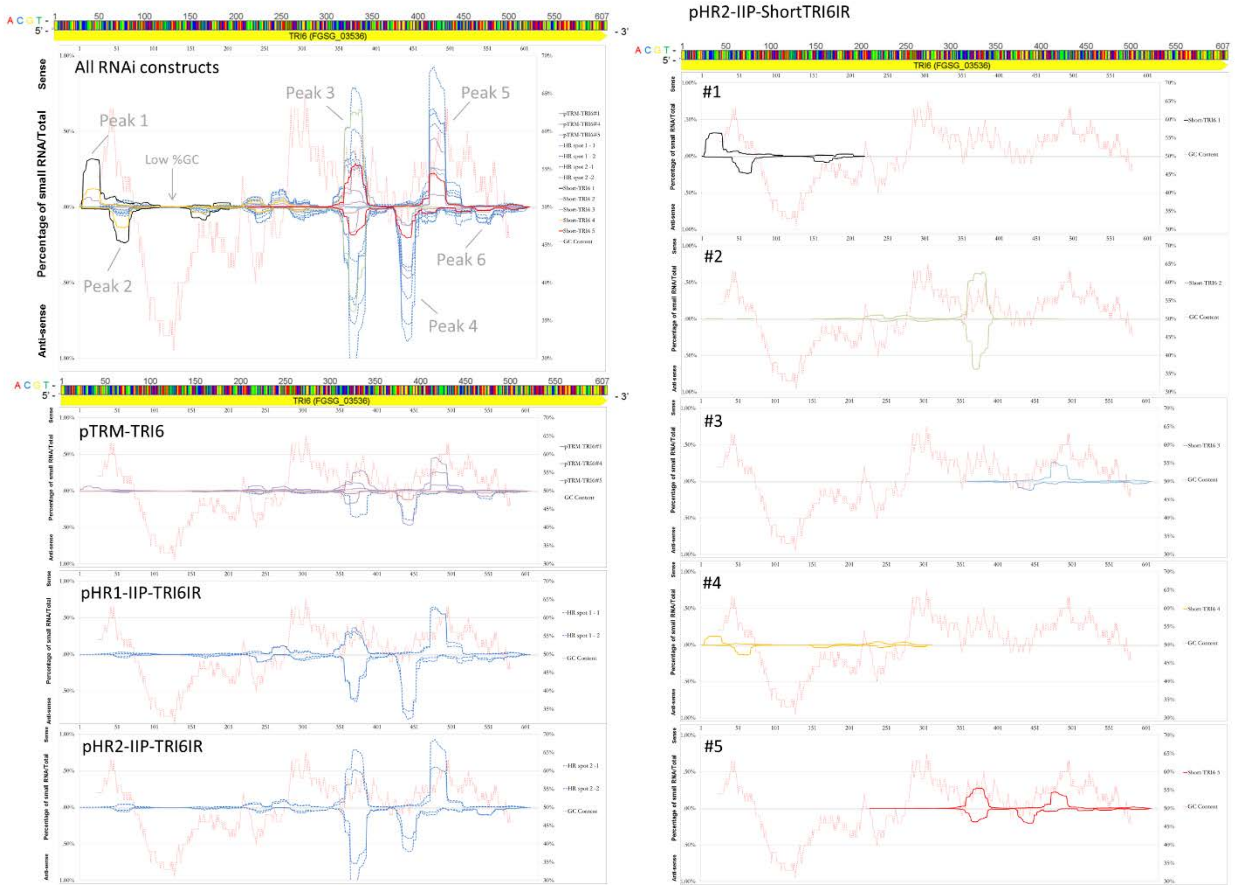

Fig O. Small RNA profiles of all mutants in this study. Small RNA profiles (map position and relative abundance) of sRNA that mapped to *TRI6* (x axis is *TRI6* from 5' – 3') in all mutants, random insertion pTRM-TRI6, site-direct pHR1-IIP-TRI6IR, pHR2-IIP-TRI6IR, and pHR2-IIP-ShortTRI6IR (1 – 5).
